# Genetically-modified activation strategy facilitates the discovery of sesquiterpene-derived metabolites from *Penicillium brasilianum*

**DOI:** 10.1016/j.synbio.2024.12.006

**Published:** 2024-12-25

**Authors:** Wenni He, Xiaoting Rong, Hui Lv, Lihua Zhang, Jinglin Bai, Lu Wang, Liyan Yu, Lixin Zhang, Tao Zhang

**Affiliations:** a Institute of Medicinal Biotechnology, Chinese Academy of Medical Sciences & Peking Union Medical College, Beijing, 100050, China; b State Key Laboratory of Component-based Chinese Medicine, Institute of Traditional Chinese Medicine, Tianjin University of Traditional Chinese Medicine, Tianjin, 301617, China; c State Key Laboratory of Bioreactor Engineering, East China University of Science & Technology, Shanghai, 200237, China

**Keywords:** Genome mining, Biosynthetic gene cluster, Sesquiterpenoids, *Penicillium brasilianum*, Meroterpenoid, Genetically-modified activation strategy

## Abstract

Genome mining has revealed that *Penicillium* spp. possess numerous down-regulated or cryptic biosynthetic gene clusters (BGCs). This finding hinted that our investigation of fungal secondary metabolomes is limited. Herein, we report a genetically-modified activation strategy to characterize the spectrum of sesquiterpenoids produced by *Penicillium brasilianum* CGMCC 3.4402. The cryptic or down-regulated pathways were stimulated by constitutive expression of pathway-specific regulator gene *berA* responsible for berkeleyacetals biosynthesis from *Neosartorya glabra*. Chemical analysis of the extracts from the mutant strain *Pb*-OE:*berA* enabled the isolation of two new compounds including one bisabolene-type arpenibisabolane C (**1**), one daucane-type arpenicarotane C (**4**), along with four known sesquiterpenoids including arpenibisabolane A (**2**), eupenicisirenins A (**3**), arpenicarotane B (**5**) and aspterric acid (**6**). The assignments of their structures were elucidated from detailed analyses of spectroscopic data, electronic circular dichroism calculation, and biogenetic considerations. The bioassay of isolated compounds (**1**–**6**) exhibited no cytotoxic activities against three tumor cells including MCF-7, HepG2, and A549. Arpenibisabolane C (**1**) and A (**2**) showed weak inhibition bioactivities on aquatic pathogens *Vibrio owensii* and *Vibrio algivorus*. Moreover, phylogenetic analysis and sequence alignments of crucial sesquiterpene synthases were performed. Based on the chemical structures and biogenetic investigations, a hypothetic pathway of new compounds (**1**, **4**) was proposed.

## Graphical abstract

**Figure:**
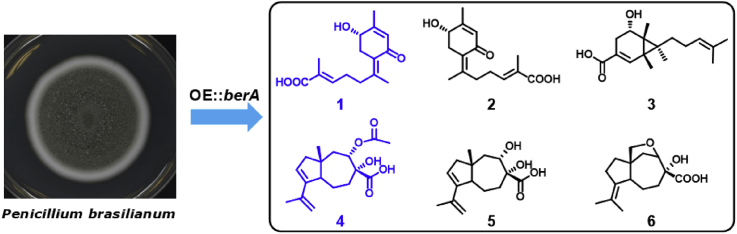

## 1 Introduction

Filamentous fungi are particularly adept at biosynthesizing a wide range of structurally complexified secondary metabolites (SMs) [1]. However, it is becoming increasingly difficult to characterize lead compounds by conventional chemical-only approaches due to the higher tendency of repeated isolation of known metabolites [2]. The fact that the biosynthetic potential has not yet been exploited is due to the observation that most BGCs are not actively expressed in general laboratory conditions [3,4]. Furthermore, genome sequencing has metamorphosed SMs discovering endeavors, demonstrating that the capacity of fungi to synthesize compounds was far more than we assumed previously [1,5,6]. To overcome these limitations, several methodologies have been conducted to activate the cryptic or down-regulated biosynthetic pathways, exemplified as "one strain many compounds (OSMAC)" approach [7,8], chemical epigenetic modification [9], coculture [10,11], metabolic engineering of targeted pathway [3,4], promoter insertion [12], and heterologous expression of gene clusters using different host cells [13,14]. Previous studies have manifested that manipulating these BGCs, particularly the activation of targeted pathways via overexpressing cluster-specific activator coding genes, is a prospective and efficient strategy to facilitate bioprospecting for new metabolites exhibiting fascinating structures [3,4]. Thus, the genetically-modified activation approach renders the mining of bioactive compounds promising and efficient.

*Penicillium* is a widely distributed and fundamentally saprophytic genus including 354 accepted species [15], and a varying assortment of metabolites exhibiting complexified structural features are isolated from *Penicillium* species [16,17]. The compounds derived from *Penicillium* strains, cover diverse bioactivities including antitumor, anticholesterolemic, antimicrobial, anti-inflammatory, and antioxidant activities, making them potential drug candidates [18]. *P*. *brasilianum* could biosynthesize compounds including verruculogen, 3,5-dimethylorsellinic acid (DMOA)-derived meroterpenoids, griseofulvin, and brasiliamides, which has been the focus of many researchers in the continuous exploiting for bioactive compounds [19]. A more detailed investigation of *P*. *brasilianum* species and metabolites generated from this strain has already been carried out and deserves to be particularly mentioned, including the isolate *P*. *brasilianum* LaBioMMI 024, the isolate FKI-3368, the isolate JV-379, *P*. *brasilianum* Batista, the isolate NBRC 6234 and the isolate LaBioMMi 136 [19]. However, for *P*. *brasilianum* species, few sesquiterpenoids have been isolated to date.

Previous studies have demonstrated that BerA served as a pathway-specific activator during the biosynthesis of meroterpenoid berkeleyacetals from the strain *N*. *glabra* [4]. In this study, we aimed to investigate the hypothesis that activation of down-regulated metabolic pathways of sesquiterpenoids could be stimulated through overexpressing the meroterpenoid pathway activator BerA in *P*. *brasilianum*. Chemical investigation of the mutant strain *Pb*-OE:*berA* facilitated the isolation of two novel sesquiterpenoids, including one bisabolene-type arpenibisabolane C (**1**) and one daucane-type arpenicarotane D (**4**), along with four known sesquiterpenoids. Herein, we reported the isolation and structural elucidation of these compounds. The hypothetical biosynthetic pathway for new sesquiterpenoids was also proposed.

## 2 Materials and methods

### 2.1 Strains and culture conditions

The fungi *P*. *brasilianum* CGMCC 3.4402 (derived from *P*. *brasilianum* NBRC 6234 but lacks the complete *prh* cluster due to some mutations) and the derived mutants *Pb*-OE:*berA* used in this study were preserved in the China Pharmaceutical Culture Collection Center (CPCC). The fungi were routinely cultivated on potato dextrose medium (PDA) and incubated at 28 °C for one week. Fermentation of mutants *Pb*-OE:*berA* and wild-type strain to produce secondary metabolites was performed using rice medium (20 g rice/20 mL of deionized water in 250 mL Erlenmeyer flask) [3,7]. To produce mycelium for seed culture, the strain was cultured in liquid MEP medium (malt extract broth 2.0 %, BD, soy bean powder 0.2 %). The culture was cultivated on a rotatory shaker (150 rpm) for 4-day. For large-scale fermentation, the mycelia were harvested and inoculated equally into fermentation medium described above. The cultures (100 g rice/100 mL water in 500 mL flask, 60 flasks) were cultivated statically for twenty-five days [20,21].

Standard DNA cloning experiments were carried out using the strain *E*. *coli Trans*-T1. *E*. *coli* culture carrying the respective plasmid was incubated in Luria-Bertani (LB) medium supplemented with kanamycin (50 μg/mL). The induction medium (IM) used for co-cultivation included K_2_HPO_4_ 2.05 g, K_2_HPO_4_·3H_2_O 1.9 g, (NH_4_)_2_SO_4_ 0.5 g, MgSO_4_·7H_2_O 0.5 g, glucose 1.8 g, 4 % (w/v) glycerol and a final concentration of 40 mM MES hydrate (Sigma–Aldrich) per liter [[22], [23], [24]]. Yeast extract broth (YEB) used for cultivating the strain *A. tumefaciens* (AGL-1) contained 1 g yeast extract, 10 g peptone, 5 g sucrose, and 0.5 g MgSO_4_·7H_2_O per liter (pH 7.2). The strains and genetically engineered cells are listed in Table 1.

**Table 1:** Strains, plasmids, and oligonucleotides used in this study.

Strains and plasmids	Description	Source
*E. coli* Trans T1	*lacх74 recA1 deoR F – mcrA Δ (mrr-hsdRMS-mcrBC) ϕ80 lacZΔM15Δ araD139Δ (ara-leu)7697 galU galK*	Transgen
*S*. *cerevisiae* BJ5464	*MATα ura3–52 trp1 leu2-Δ1 his3-Δ200 pep4::HIS3 prb1-Δ1.6R can1 GAL*	[13,18]
*Agrobacetrium tumefaciens* AGL-1	carrying pCAMBIA3301 plasmid, Kan^R^	[3,26]
*P*. *brasilianum* CGMCC 3.4402	Wild type strain	CGMCC
*T1*-OE::*berA*	*E.* coli carrying overexpression cassette OE:*berA*	This study
BJ5464-OE::*berA*	*BJ5464* cell carrying OE::*berA*	This study
AGL-1-OE::*berA*	AGL-1 cell carrying OE::*berA*	This study
*Pb*-OE::*berA*-R3	*berA* overexpression mutant of *P***.***brasilianum*	This study
*Pb*-OE::*berA*-R7	*berA* overexpression mutant of *P***.***brasilianum*	This study
YET	Cloning plasmid of yeast containing TRP1 marker	[13,18]
pAg1-H3	Cloning plasmid for ATMT containing Hyg + marker	[3,18]
pPB01	YET vector carrying OE::*berA* cassette	This study
pPB02	pAg1-H3 vector carrying OE::*berA* cassette	This study
**Oligonucleotides**	**Sequence (5′-3′)**	**Purpose**
Z01 (PgpdA-F)	actatcaactattaactatatcgtaataccatATGGGGCCCGGTACCGAATTCCCTTGTATCTCT	*PgpdA* amplification
Z02 (PgpdA-R)	AGTCGGGTACCGTGCGTGCCGCAGCCATgggtgatgtctgctcaagcggggtagct	
Z03 (berA-F)	agctaccccgcttgagcagacatcacccATGGCTGCGGCACGCACGGTACCCGACT	*berA* amplification
Z04 (*berA*-R)	tgaaaactataaatcgTGAAGGCATGTTTAAACATTTAAATATACTGTCTTGTTTGTCG	
Z05 (cha-*hyg*-F)	TCGACAGAAGATGATATTG	Hyg + gene characterization
Z06 (cha-*hyg*-R)	AAGAAGGATTACCTCTAA	
Z07 (rt-*tub*-F)	GAGGTTGAGGACCAGATGC	real-time PCR of tubulin gene
Z08 (rt-*tub*-R)	CCAATACGCTTGAACAGCT	
Z09 (rt-*carA*-F)	CATAATCCTGACCTGGCTCT	real-time PCR of gene *carA*
Z10 (rt-*carA*-R)	CTTGCACCTGCATTGTTG	
Z11 (rt-*bisA*-F)	TACTCTTTGGCTCTTCTGCT	Real-time PCR of gene *bisA*
Z12 (rt-*bisA*-R)	GATGTCGCTGGTTGTTGA	

### 2.2 Gene synthesis and vector construction

The gene *berA* from the strain *N*. *glabra* was synthesized by Genscript for heterologous expression in *Penicillium* [4]. The oligonucleotide primers used for amplifying the genes are listed in Table 1. PCR reactions were conducted using Phusion® High-Fidelity DNA Polymerase (New England Biolabs) based on the manufacturer's instructions. The strong constitutive PgpdA promoter coding gene (glyceraldehydes-3-phosphate dehydrogenase promoter) of *Aspergillus nidulans* was obtained by PCR amplification in which the plasmid pRF-HUE serves as DNA template [23]. PCR amplification of the gene *berA* was performed using primers (Z03/Z04) listed in Table 1. The preparation of yeast competent cells and the following transformation were conducted using Frozen-EZ Yeast Transformation II kit (Zymo Research) according to the manufacturer's procedure. The two PCR amplicons (*berA* and *PgpdA*) were transformed into *Saccharomyces cerevisiae* BJ5464 cells simultaneously with a linearized YET vector (TRP1 marker) to create the plasmid pPB01 [4,25] (Table 1).

Yeast plasmids were extracted using a E.Z.N.A® Yeast Plasmid Miniprep Kit (Solarbio, Beijing, China) and used for transforming chemically competent *E. coli* Trans1-T1 cells (TransGen, Beijing, China). Plasmid DNA was extracted from the positive transformant cultivated in LB medium supplemented with ampicillin using an E.Z.N.A® Plasmid DNA Mini Kit (Omega) and further verified by DNA sequencing. Next, The DNA fragment carrying *berA* and *PgpdA* was further digested with *Pme*I and *Apa*I, and the recovered ∼4.7 kb DNA fragment was ligated into the linearized vector pAg1-H3 prepared elsewhere [3,18]. The plasmids pPB02 in the positive transformant screened by diagnostic PCR were sequenced for verification and further transformed into *A*. *tumefaciens* AGL-1 cells (Table 1).

### 2.3 ATMT transformation of *P*. *brasilianum*

The *Agrobacterium*-mediated transformation (ATMT) of the strain *P*. *brasilianum* was conducted as per published protocols [18,23,24]. In brief, *A. tumefaciens* AGL-1 cells were transformed with overexpression plasmid pPB02 by electroporation. The transformed AGL-1 cells were subsequently cultured in YEB broth supplemented with kanamycin (50 μg/mL) for 48 h at 30 °C. One milliliter of the cell culture was harvested and washed using induction medium (IM). Bacterial cells were diluted in IM broth (5 mL) with 200 μM acetosyringone (Sigma-Aldrich) to an OD_600_ = 0.2 and then incubated (200 rpm, 30 °C) until an OD_600_ = 0.7 was reached.

Fresh spores of *P*. *brasilianum* were harvested on PDA plates for 7 days at 28 °C and passed through Cell Strainer (Falcon) to afford conidia suspension. Conidia were counted microscopically, and adjusted to 1 × 10^7^ cells/mL in IM broth. Aliquots of 100 μL AGL-1 cells carrying the plasmid pPB02 were mixed with an equal volume of spore suspension and were spread onto sterilized nitrocellulose membranes (Cat. no. D9527, Sigma-Aldrich) placed on IM agar plates supplemented with 200 μM acetosyringone [3,24]. The co-cultivation experiments were conducted at 28 °C for 48 h [26]. The membranes were then transferred to selection PDA plates containing 100 μg/mL hygromycin and 300 μg/mL cefotaxime. The putative transformants were subsequently transferred to new selection plates and incubated at 28 °C for 3 days. Primers used for PCR screening are listed in Table 1.

### 2.4 Chemical reagents and chemical analysis

NMR solvents were purchased from Sigma-Aldrich. Silica gel (SiO_2_, 200–300 mesh) for column chromatography and silica GF254 were purchased from Qingdao Marine Chemical Company. Sephadex LH-20 (GE Healthcare) and ODS (YMC Corporation) were used for column chromatography. High-performance liquid chromatography (HPLC) analyses were achieved on an Agilent 1290 instrument using an Agilent- Eclipse SB-C18 column (4.6 × 250 mm, 5 μm). The medium-pressure liquid chromatography was performed on Combi Flash Rf 200 (Teledyne Isco, Lincoln NE, USA) with a SEPAF FLASH® Flash silica gel column (40–63 μm, 60 Å, 330 g, Santai Technologies, China). TLC was carried out on silica gel GF254 plates. The semi-preparative HPLC (pHPLC) separations were conducted on an Agilent 1200 apparatus allocated with a DAD detector using an Agilent-SBC18 column (9.4 × 250 nm, 5 μm). Nuclear magnetic resonance (NMR) spectra were collected from a Bruker AVIII-600 spectrometer with TMS serving as internal standard (150 MHz for ^13^C NMR and 600 MHz for ^1^H NMR, Bruker Corporation, Germany), which were dissolved in DMSO or CDCl_3_. Circular dichroism spectra were recorded on a Jasco J-815 spectropolarimeter (Jasco, Tokyo, Japan). High-resolution electrospray ionization mass spectra (HR-ESI-MS) analysis was measured using a Thermo LTQ Orbitrap XL Mass Spectrometer installed with an electrospray ionization source in negative ion mode (Thermo Fisher Scientific, CA, USA).

### 2.5 Extraction and isolation of sesquiterpenoids

The fermented material of mutant *Pb*-OE:*berA*-R3 was harvested and ultrasonically extracted with ethyl acetate (EtOAc) 3 times (each time for 2 h) [20,21]. The organic layer was evaporated to dryness and yielded a crude residue (*ca.* 39 g) after EtOAc recovery. The crude extract was separated on a silica gel (200–300 mesh) column chromatography, eluting with CHCl_2_/MeOH (v/v, 100:0 → 95:5 → 90:10 → 85:15 → 80:20 → 75:25 → 70:30 → 65:35 → 60:40 → 55:45 → 50:50 → 45:55 → 40:60 → 0:100, 15 mL/min) to obtain fifteen fractions (A1-A15) based on TLC analysis. Further HPLC-UV analysis of each fraction showed that fraction A5 might contain the putative-induced metabolites. Fraction A6 (16.0 g) was further separated by an ODS column eluting with acetonitrile-H_2_O (containing 0.01 % TFA) (25:75 → 100:0) to generate fifteen subfractions (B1 to B15). Subfraction B5 was purified by pHPLC eluting with acetonitrile-H_2_O (v/v, 20:80; flow rate 4.0 mL/min, column temperature 35 °C) to give compound **1** (6.7 mg, *t*_*R*_ = 21.0 min), and **2** (5.2 mg, *t*_*R*_ = 24.0 min). Subfraction B9 was purified using pHPLC eluting with acetonitrile-H_2_O (0.01 % TFA) (v/v, 30:70; flow rate 4.0 mL/min, column temperature 35 °C) to afford compound **3** (4.0 mg, *t*_*R*_ = 37.0 min). Then, subfraction B11 was separated by pHPLC with acetonitrile-H_2_O (0.01 % TFA) (v/v, 40:60; flow rate 4.0 mL/min, column temperature 35 °C) to give compound **4** (10.0 mg, *t*_*R*_ = 29.0 min) and **5** (5.4 mg, *t*_*R*_ = 25.8 min). Purification of the subfraction B13 was performed by pHPLC, eluting with acetonitrile-H_2_O (0.01 % TFA) (v/v, 45:55; flow rate 4.0 mL/min, column temperature 35 °C) to give compound **6** (12.9 mg, *t*_*R*_ = 8.0 min).

### 2.6 RNA extraction, purification, and real-time PCR analysis

The RNAs from fermentation samples including the mutant *Pb*-OE:*berA* and wild-type strain were extracted using PureLink™ RNA Mini Kit (Invitrogen, USA) based on the standard protocol. Removals of gDNA were achieved by treatment with RNase-free DNase I (Takara). Recovery of RNA was carried out using *EasyPure*® RNA purification kit (TransGen). The RNA concentration was quantified using a Nanodrop 2000/2000c spectrometer (Thermo Fisher Scientific) and the RNA integrity was identified by electrophoresis. The first-strand cDNA synthesis was obtained from 500 ng total RNA with HiScript reverse transcriptase (Vazyme) using oligo (dT) primer (Takara) following the manufacturer's instruction. Real-time PCR was conducted using Phanta Max Super-Fidelity DNA polymerase (Vazyme Biotech) using 25 ng of reverse-transcribed RNA [3]. Primers are listed in Table 1. The relative quantification of mRNAs was normalized to the levels of the beta-tubulin gene based on the 2^−*ΔΔCT*^ method [3,27]. Data are calculated from the average value from triplicate tests.

### 2.7 Cell viability assay

All the metabolites were tested for their cytotoxic activities as previously reported methods [7,18]. The cells including MCF7, A549, and HepG2 were treated with gradient concentrations of isolated metabolites for 48 h (DMSO and cisplatin were used as the negative and positive control, respectively). The cytotoxicity was measured using the CCK-8 method [28]. Ten *μ*L of CCK-8 solution was added to each well of the plate and then the plate was incubated for 4 h. Measuring the absorbance at 450 nm and 630 nm using a microplate reader (Bio-Rad, Fitchburg, WI, USA). All experiments were repeated thrice in three wells of the microplate. Purities of all tested compounds were >95 % detected by HPLC-ELSD.

#### 2.7.1 Antimicrobial assay

The microplate assay was used in the antimicrobial evaluation of isolated metabolites (**1**–**6**) against the aquatic pathogens including *Vibrio algivorus* CGMCC 1.16112 and *V*. *owensii* CGMCC 1.8700. Two sterile 96-well plates were labeled and a volume of tested compounds in 5 % (v/v) DMSO was pipetted into the first row of the plate [29]. To all other wells 50 μL of Mueller-Hinton broth (Sigma) was added. Serial dilutions (final concentrations of compounds: 128, 64, 32, 16, 8, 4, 2, 1, 0.5, 0.25 μg/mL) were performed using a multichannel pipette. Then, 10 μL of bacterial suspension were inoculated to achieve a final concentration of ∼5 × 10^5^ CFU/mL. Chloramphenicol serves as a positive control [30]. Plates were covered with the seal and incubated at 37 °C for 18 h, minimal inhibition concentrations (MICs) were determined when no visible growth was observed. The average of three values was calculated.

## 3 Results

### 3.1 PgpdA promoter-controlled overexpression of *berA* induces SMs yielding

**Fig. 1:**
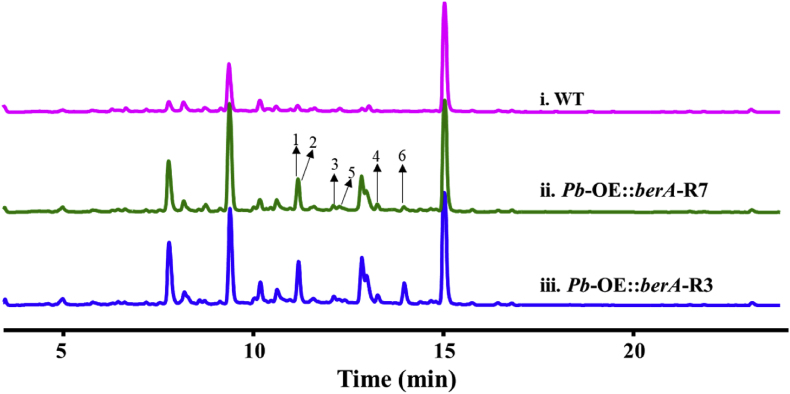
HPLC profiles of organic extracts obtained from the wild-type strain *P. brasilianum* and OE:*berA* mutants. (i) Wild-type strain; (ii) *Pb-*OE:*berA*-R7; (iii) *Pb-*OE:*berA*-R3. The extracts were analyzed by measuring UV absorbance spectra at 230 nm on an Agilent 1290 Infinity system equipped with an Alltech ELSD 2000 detector.

Previous studies indicated that the transcriptional regulator BerA has been verified that could trigger up-regulation of the biosynthetic locus responsible for berkeleyacetals biosynthesis [4]. To further prove the concept that constitutive overexpression of the encoding gene *berA* (accession number, PQ723751) could lead to the activation of potential uncharacterized BGCs in *P*. *brasilianum*, the *berA* was synthesized from GenScript. Then, the gene berA and *PgpdA* promoter were amplified using plasmid containing synthesized berA and pRF-HUE as templates, respectively [24]. Yeast-based homologous recombination was performed to construct pPB01 plasmid, in which the linearized pYET serves as a vector. The recovered expression cassette OE: *berA* was ligated into the linearized pAg1-H3 vector to generate the pPB02 plasmid. Using the *A*. *tumefaciens*-mediated transformation (ATMT) approach, *P*. *brasilianum* was transformed with the plasmid pRB02 harboring *berA* under the control of the constitutive glyceraldehyde-3-phosphate dehydrogenase promoter *PgpdA* from *A. nidulans*. Among forty-six hygromycin-resistant transformants, ten were randomly selected, sub-cultured, and PCR identified for T-DNA insertion using the resistance gene (*hyg*^+^) and *berA* gene. All the hyg^+^ transformants displayed a 2-kb amplicon against *hyg*^+^ gene and a 1.5-kb amplicon against *berA* gene, confirming the integration of T-DNA into the *P*. *brasilianum* genome. Two representative mutants were selected and designated as *Pb*-OE:*berA*-R3, and *Pb*-OE:*berA*-R7 (Fig. S1, supporting information). Finally, we tested whether the *berA* gene could alter the metabolite profiling in strain *P*. *brasilianum*. As a result, HPLC analysis revealed that the mutants *Pb*-OE:*berA*-R3 and *Pb*-OE:*berA*-R7 produce more compounds than wild-type strain (Fig. 1). In contrast, poor and lower productivity of SMs of parental strain was observed. These results indicated that the exogenous activator coding gene *berA* was expressed, confirming that BerA could be utilized to promote the generation of secondary metabolites in *P*. *brasilianum*.

### 3.2 Characterization of induced metabolites from the mutant strain *Pb*-OE::*berA*

**Fig. 2:**
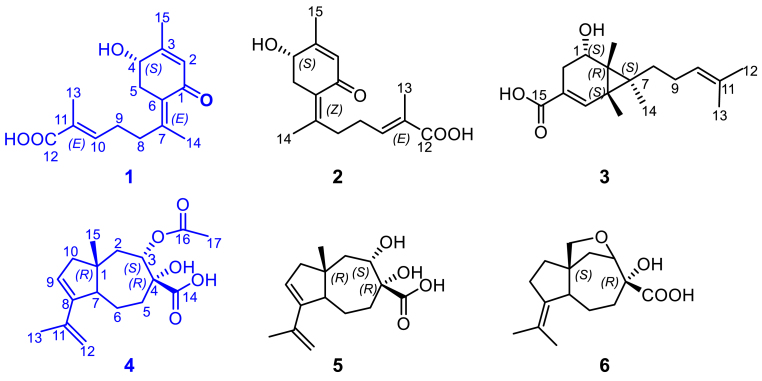
The chemical structures of compounds **1**–**6**, isolated from *P*. *brasilianum*-derived mutant *Pb-*OE:*berA*-R3.

**Fig. 3:**
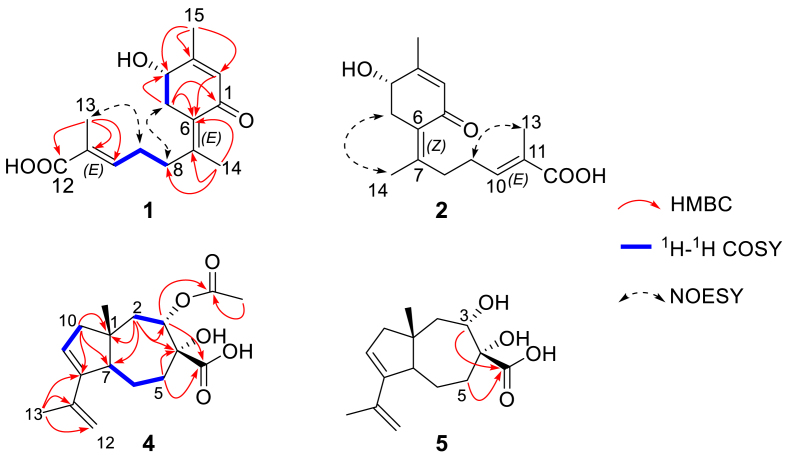
Key HMBC, ^1^H–^1^H COSY and NOESY correlations of compounds **1**, **2, 4** and **5**.

**Fig. 4:**
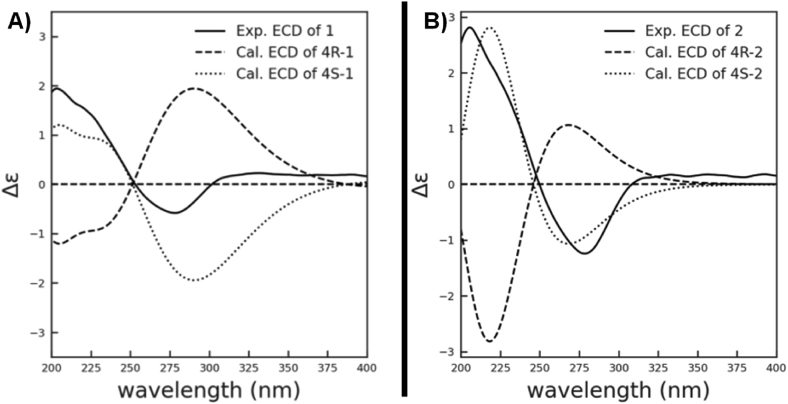
Experimental and calculated ECD spectra of compounds **1** and **2**. A) arpenibisabolane C (**1**); B) arpenibisabolane A (**2**).

To get sufficient amounts of compounds for full structure elucidation, a large-scale cultivation of the mutant strain *Pb*-OE:*berA*-R3 was performed, which led to the isolation of two new sesquiterpenoids **1** and **4**, along with four known ones, *i*.*e*., **2**–**3** and **5**–**6** (Fig. 2). Compound **1** was obtained as colorless oil, ${\left[\alpha \right]}_{D}^{25}$ 40 (*c* 0.02, MeOH), UV (MeOH) *λ*_max_ (log *ε*) 225 (5.23), 285 (2.12) nm. Its molecular formula was established as C_15_H_20_O_4_ based on HRESIMS data, [*m*/*z* 265.14272 [M+H]^+^ (calcd for C_15_H_21_O_4_ 265.14344)] corresponding to six degrees of unsaturation. The 1D NMR (Table 2) and HSQC data of **1** revealed the presence of 15 carbon signals in this compound, including two carbonyl carbons at *δ*_C_ 193.3 and 171.4, four quaternary sp^2^ carbons at *δ*_C_ 164.2, 148.9, 129.8, and 128.9, two sp^2^ methines (*δ*_C_/*δ*_H_ 142.1/6.77 and 129.6/5.82), one oxygen-bearing methine (*δ*_C_/*δ*_H_ 70.1/4.25), three methenes (*δ*_C_/*δ*_H_ 38.5/3.01, 2.59, 35.8/2.39, and 28.1/2.40) and three methyls (*δ*_C_/*δ*_H_ 21.2/2.07, 20.2/2.01, and 12.4/1.81) (Table 2). The six-membered ring system in **1** was determined on the basis of the HMBC correlations of H_3_-15 (*δ*_H_ 2.01) with C-2 (*δ*_C_ 129.6), C-3 (*δ*_C_ 164.2) and C-4 (*δ*_C_ 70.1), H_2_-5 (*δ*_H_ 2.59, 3.01) with C-1 (*δ*_C_ 193.3), C-4 (*δ*_C_ 70.1), and C-6 (*δ*_C_ 128.9), and H-2 (*δ*_H_ 5.82) with C-6 (*δ*_C_ 128.9), coupled with a ^1^H–^1^H COSY correlation between H-5 and H-4. The ^1^H–^1^H COSY correlations between H-8/H-9 and H-9/H-10, along with the HMBC correlations from H_3_-13 (*δ*_H_ 1.81) to C-10 (*δ*_C_ 142.1), C-11 (*δ*_C_ 129.8), and C-12 (*δ*_C_ 171.4), and H_3_-14 (*δ*_H_ 2.07) to C-7 (*δ*_C_ 148.9), and C-8 (*δ*_C_ 35.8) established a side chain as a 2-methyl hepta-2-enoic acid substructure (Fig. 3). The HMBC correlations from H_3_-14 (*δ*_H_ 2.07) to C-6 revealed the connection of C-6 and C-7. The spectroscopic data of **1** (Fig. 2, Figs. S3–S9, Table S1, supporting information) are almost identical with **2** reported for arpenibisabolane A (Fig. S2, Fig. S18-S24, Table S1, supporting information) [[31], [32], [33]]. The double bonds Δ^6, 7^ and Δ^10, 11^ were assigned as E-configured based on the NOE correlations between H-8 (*δ*_H_ 2.39)/H-5 (*δ*_H_ 3.01) and H_3_-13 (*δ*_H_ 1.81)/H-9 (*δ*_H_ 2.40) which was different from the Z-configuration of Δ^6, 7^ in **2**. The absolute configuration of C-4 was determined as 4*S* by ECD calculation and comparison (Fig. 4). It was named as arpenibisabolane C.

**Table 2:** ^1^H and ^13^C NMR data for compounds **1** and **4** in CD_3_OD.

	1		4	
Pos.	*δ*_C_	*δ*_H_	*δ*_C_	*δ*_H_
1	193.3		44.4	
2	129.6	5.82 (1H, m)	42.8	1.94 (2H,m)
3	164.2		78.1	5.26(1H,t,*J* = 8.4)
4	70.1	4.25 (1H, m)	80.9	
5	38.5	3.01 (1H, m), 2.59 (1H, m)	32.3	2.11(1H,m),
				1.89(1H,m)
6	128.9		23.3	2.15(1H,m),
				1.35(1H,m)
7	148.9		51.1	3.35(1H, m)
8	35.8	2.39 (2H, m)	149.8	
9	28.1	2.40 (2H, m)	125.8	5.57 (1H, d, *J* = 2.4 Hz)
10	142.1	6.79 (1H, m)	49.2	2.16(1H,m), 2.02(1H,m)
11	129.8		142.1	
12	171.4		111.9	4.84(1H, m),
				4.79(1H,m)
13	12.4	1.81 (3H, s)	22.6	1.84 (3H, s)
14	21.2	2.07 (3H, s)	172.4	
15	20.2	2.01 (3H, s)	20.0	1.09 (3H, s)
16			171.7	
17			21.1	1.97(3H, s)

**Fig. 5:**
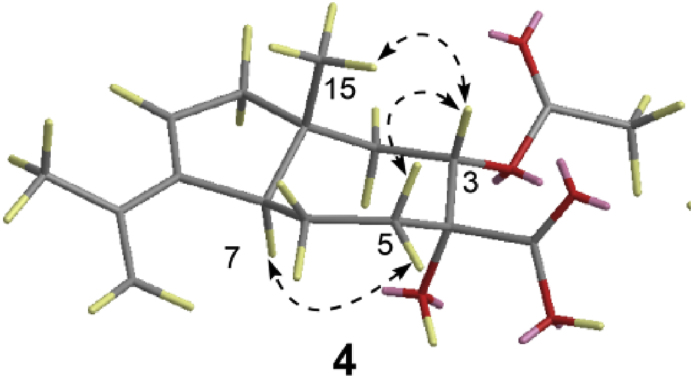
Key nuclear overhauser spectroscopy (NOESY) correlations of compound **4**.

Compound **4** was obtained as colorless oil, ${\left[\alpha \right]}_{D}^{25}$ 40 (*c* 0.02, MeOH), UV (MeOH) *λ*_max_ (log *ε*) 240 (4.54) nm. Its molecular formula was established as C_17_H_22_O_5_ based on HRESIMS data, [*m*/*z* 307.15393 [M+H]^+^ (calcd for C_17_H_23_O_5_ 307.15510)] corresponding to six degrees of unsaturation. A detailed analysis of its 1D NMR (Table 2) and HSQC data exhibited the presence of 17 carbon signals in this compound, including two carbonyl carbons at *δ*_C_ 172.4 and 171.7, two quaternary sp^2^ carbons at *δ*_C_ 149.8 and 142.1, one sp^2^ methine (*δ*_C_/*δ*_H_ 125.8/5.57), one terminal olefinic carbon (*δ*_C_/*δ*_H_ 111.9/4.84, 4.79), one oxygen-bearing full substituted carbon at *δ*_C_ 80.9, one oxygen-bearing methine (*δ*_C_/*δ*_H_ 78.1/5.26), one methane (*δ*_C_/*δ*_H_ 51.1/3.35), one quaternary carbon at *δ*_C_ 44.4, four methenes (*δ*_C_/*δ*_H_ 49.2/2.16 and 2.02, 42.8/1.94, 32.3/2.11, 1.89, and 23.3/2.25, 1.35), and three methyls (*δ*_C_/*δ*_H_ 22.6/1.84, 21.1/1.97, and 20.0/1.09). The ^1^H–^1^H COSY correlations of H-2/H-3, H-5/H-6 and H-6/H-7 corresponded to –C-2–C-3– and –C-5–C-6–C-7– systems, coupled with the HMBC correlations of H-2 (*δ*_H_ 1.94) with C-1 (*δ*_C_ 44.4), C-3 (*δ*_C_ 78.1), C-4 (*δ*_C_ 80.9), and C-7 (*δ*_C_ 51.1), and H-5 with C-4 (*δ*_C_ 80.9) confirmed the presence of heptatomic ring with two oxygen-bearing carbons at C-3 and C-4 (Fig. 3). Meanwhile, the ^1^H–^1^H COSY correlation of H-9/H-10 and the HMBC correlations of H-10 with C-1 (*δ*_C_ 44.4), C-7 (*δ*_C_ 51.1), and C-8 (*δ*_C_ 149.8) established the cyclopentane substructure of **4**. In addition, the HMBC correlations of H_3_-13 (*δ*_H_ 1.84) with C-8 (*δ*_C_ 149.8), C-11 (*δ*_C_ 142.1), and C-12 (*δ*_C_ 111.9) provided evidence for the presence of the isopropenyl group. These data indicated that compound **4** was similar to **5**, except for an additional acetoxy group [33]. Furthermore, the HMBC correlations of H-3 (*δ*_H_ 5.26) with C-14 (*δ*_C_ 172.4) and C-16 (*δ*_C_ 171.7), H-5 (*δ*_H_ 1.89) with C-14 (*δ*_C_ 172.4), and H_3_-17 (*δ*_H_ 1.97) with C-16 (*δ*_C_ 171.7) revealed the presence of an ethyl ester of 3-hydroxy group and a carboxyl group at C-4. Thus, the planar structure of **4** was established (Fig. 2). The relative configuration of **4** was elucidated using NOESY correlations of H-3 (*δ*_H_ 3.72)/H_3_-15 (*δ*_H_ 1.06), H-3 (*δ*_H_ 3.72)/H-2*β* (*δ*_H_ 1.98) and H-2*α* (*δ*_H_ 2.08)/H-7(*δ*_H_ 2.78), which revealed the *trans*-relationship of H-7 with H-3 and H_3_-15 (Fig. 5, Figs. S10–S16, Table S1, supporting information). The relative configuration of C-4 could not be determined due to a lack of available NOESY correlations. Moreover, the ECD experimental data (Fig. S2, supporting information) of **4** was consistent with that of arpenicarotane B showing a positive cotton effect, which was characterized as a new compound in our previous co-culture study [33], indicating that the absolute configuration of **4** was determined as 1*R*,3*S*,4*R*,7*R*. It was named as arpenicarotane C.

The remaining known compounds were identified as arpenibisabolane A (**2**) [33], eupenicisirenins A (**3**) [34], arpenicarotane B (**5**) [33], aspterric acid (**6**) [35,36] by comparison of their NMR spectral data with those reported in the literature (Figs. S17–S34, supporting information). Among them, compounds **2** and **5** were also characterized in our previous coculture research. Detailed structural elucidation of these compounds is described in the supporting information file.

### 3.3 Bioinformatic mining and real-time PCR analysis of key STSs encoding genes

**Fig. 6:**
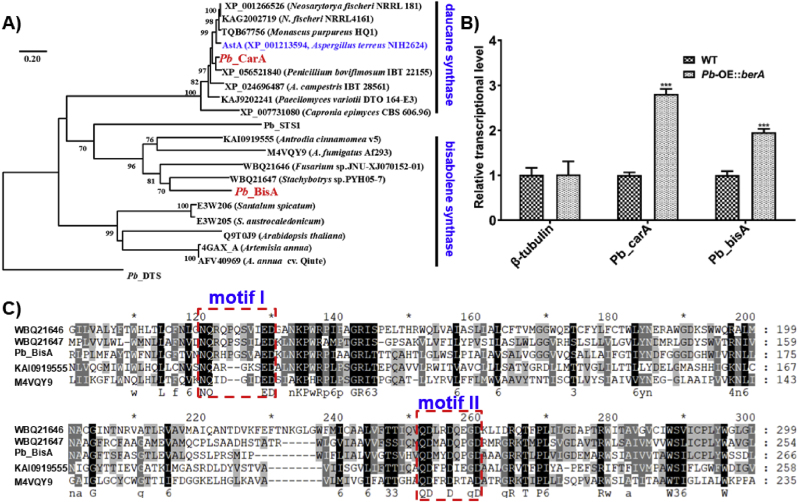
Bioinformatic analysis of sesquiterpene synthases and real-time analysis of involving biosynthetic STSs-coding genes. A) Phylogenetic tree of sesquiterpene synthases from *P. brasilianum*, characterized STSs or homologues using the Neighbor-Joining method. The scale shows changes per site; numbers at branches are bootstrap values. *Pb*-CarA, *Pb*-BisA and *Pb*_STS1 are recovered from *P. brasilianum*. B) The transcription comparison of STSs-coding genes *Pb*_*carA* and *Pb*_*bisA* between the wild-type strain and the mutant *Pb-*OE:*berA*-R3. The relative abundance of mRNAs was normalized against level of the actin and β-tubulin coding gene. The error bars indicate the standard deviations from three independent experiments (∗∗∗ represents P value < 0.001). C) Alignment of amino acid sequences of the two conserved aspartate-rich motifs of *Pb*_BisA and the motifs of close UbiA-like prenyltransferases.

All six isolated compounds (**1**–**6**) are sesquiterpene-derived, which further indicates the sesquiterpene synthases (STSs) are essential for biosynthesizing these metabolites. In combination with antiSMASH bioinformatic analysis and local BLAST mining, three hypothetical STSs encoded by the fungus *P*. *brasilianum* are retrieved [37]. Therefore, a phylogenetic tree of STSs obtained from the starting fungus, as well as previously characterized *α*-bisabolol synthases and daucane synthases or homologues was constructed. In this tree, one STS designated *Pb*_CarA, *Pb*_BisA, and *Pb*_STS1 exhibited a clear separation of sesquiterpene synthases (Fig. 6A). *Pb*_CarA formed a distinct branch with AstA from *Aspergillus terreus* NIH 2624 and sesquiterpene cyclase homologues. STSs of this subfamily are crucial factors involved in the biosynthesis of ducane (carotene)-derived metabolites, exemplified as aspterric acid and derivatives [38]. Markedly, the synthase named *Pb*_BisA formed a subclade and clustered closely with UbiA-type *α*-bisabolol synthases (BibS) from *Stachybotrys* sp. PYH05-7 and *Fusarium* sp. JNU-XJ070152 [39], *β*-trans bergamotene synthase from *A*. *fumigatus* Af293 [40], (+)-(*S*, *Z*)-*α*-bisabolene from basidiomycete *Antrodia cinnamomea* [41]. In addition, plant-derived *α*-bisabolol synthases including SspiBS from *Santalum spicatum* [42], *S*. *austrocaledonicum* or homologues from *Arabidopsis thaliana* and *Artemisia annua* are also congregated [43,44]. This further indicates the *Pb*_BisA is crucial in biosynthesizing *γ*-bisabolene-derived arpenibisabolanes.

Moreover, real-time PCR analysis showed that the transcription level of two potential biosynthetic clusters (*Pb*_*car* and *Pb*_*bis*, accession numbers are PP700700 and PP700701, respectively) in mutant *Pb*-OE:*berA*-R3 is much higher than that in wild type strain, in which two sesquiterpene synthase encoding genes (*Pb*_*carA* and *Pb*_*bisA*) were selected for detection. As a result, the transcriptional levels of the genes *Pb*_CarA and *Pb*_BisA increased 7.1-fold and 3.9-fold, respectively (Fig. 6B). The two STSs genes were proposed to be responsible for the generating skeletons of sesquiterpene-derived metabolites. Notably, the HPLC couple diode array revealed that the induced mutant yields more compounds than the wild-type strain (Fig. 1). Naturally occurring bisabolene derivatives and biosynthetic mechanisms have attracted increasing interest from research communities due to their unique architectures and biological activities. The UbiA superfamily of intramembrane prenyltransferases bears two conserved aspartate-rich motifs NXXX(G/A)XXXD (motif I) and QDXXDXXXD (motif II), which coordinate the Mg^2+^ ions that interact with the diphosphate of geranyl pyrophosphate or geranyl *S*-thiolodiphophate [41]. To detect the catalytic motifs of *Pb*_BisA, multiple sequences alignment with homologues was conducted revealing the presence of motif I as **N**_95_QRHP**G**_100_SVAE**D**_105_ and motif II as **QD**_219_MY**D**_222_QPG**D**_226_ (Fig. 6C). Moreover, protein modeling prediction using Phyre2 web portal (based on template 6M34, which belongs to digeranylgeranylglyceryl phosphate synthase, a UbiA family protein from *Methanocaldococcus jannaschii* DSM 2661) [45] further revealed the coordination of three aspartate residues for Mg^2+^ binding on conserved motif II.

### 3.4 Proposed metabolic pathway of induced representative sesquiterpenoids

**Scheme 1:**
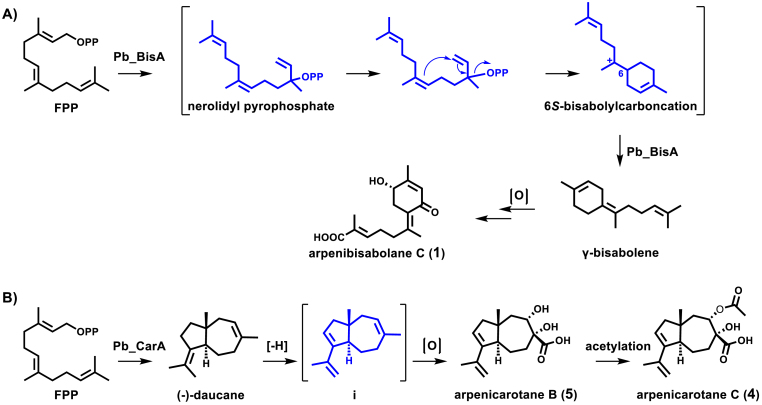
Hypothetical metabolic pathway of arpenibisabolane C and arpenicarotanes.

Compounds **1** and **2** were characterized as *γ*-bisabolene derived by comparing the chemical structure and the newly identified fungal (*Z*)-*γ*-bisabolene synthase *Pb*_BisA was involved in the biosynthesis of this class of sesquiterpenoids [46]. Nevertheless, only a few reports have been conducted to investigate the biosynthetic process of such skeletal peculiar metabolites [39]. Recently, Lin and colleagues characterized two cryptic (+)-(*S*, *Z*)-*α*-bisabolene synthases Tps1A and Tps2A from *A*. *cinnamomea*. These UbiA-type prenyltransferases could produce diverse bisabolenoids and the pioneering biogenesis investigations were limited [41]. To understand the possible biogenetic origin, their plausible biosynthetic pathway was presented in Scheme 1A. Arpenibisabolanes (**1**, **4**) initiate from farnesyl pyrophosphate (FPP), and the enzymatic cyclization generates the intermediate bisabolene. Interestingly, two chiral putative intermediates nerolidyl diphosphate (NPP) and the bisabolyl cation (6*S*-bisabolylcarboncation) might be formed during the conversion process. The wide occurrence of monocyclic bisabolene is likely connected to the simple biosynthesis from FPP featuring the common bisabolyl cation intermediate after 1,6-cyclization [41,44] (Scheme 1). For this cyclization, an isomerization conversion process of the (*E*)-configured double bond in FPP to the (*Z*)-configured double bond in bisabolyl cation intermediate is needed. Considering the isolated molecules **1** and **2** that possess highly oxygenated modifications, we tend to believe that a cascade of oxidations might undergo following the formation of bisabolene. In this scheme, arpenibisabolane C (**1**) is probably catalyzed by a multifunctional cytochrome P450 (*Pb*_BisB) [33]. Similar cytochrome P450-driven oxygenative conversions have been reported in fumagillin biosynthesis from *Aspergillus fumigatus* [40].

Biogenetically, arpenicarotanes (**4**–**5**) exhibit a unique "5 + 7" daucane skeleton. In 2018, Tang and coworkers reconstituted the metabolic pathway of aspterric acid in *A*. *terreus* (**6**) and manifested that carotane synthase AstA and two cytochrome P450s (AstB and AstC) collaboratively catalyzed the formation of compound **6** [38]. As outlined in Scheme 1B, the starter precursor FPP could be transformed into bicycle intermediate (−)-daucane through two-step cyclization [47]. The enzymatic process includes the cyclization of the acyclic tertiary pyrophosphate into the cycloheptenyl ion species and the formation of the cyclopentane ring to generate the tertiary carbonium ion species (Scheme 1B). The generation of arpenicarotane B, in which the double hydroxylation at C-3 and C-4, could be accommodated by allowing the formation of the carboxyl group at C-14, and dehydrogenation at C8–C9. These incorporating process could be performed by two cytochrome P450s (*Pb*_CarB and *Pb*_CarC) encoded in the biosynthetic gene cluster *Pb*_*car* [33]. These two P450s exhibited higher identities with amino acid sequences of AstB and AstC, respectively [33]. The final product arpenicarotane C could be generated from arpenicarotane B, in which the hydroxyl group at C-3 was acetylated. The results indicated that the class of daucane-derived sesquiterpenoids is diverse and multiple modifications occurred.

### 3.5 Biological activities of isolated sesquiterpenoids (1–6)

These compounds (**1**–**6**) were evaluated for their cytotoxic activities against MCF-7, A549, and HepG2 cell lines *in vitro* with cisplatin as the positive control. All six compounds have no obvious inhibition activities against three cell lines (Table S2, supporting information). The bisabolene derivatives **1** and **2** exhibited weak inhibitory activity against aquatic pathogens *V*. *owensii* and *V*. *algivorus* in the assay. While compounds **3**–**6** have no visible inhibition bioactivities on the tested bacterial pathogens (Table S3, supporting information).

## 4 Discussion

To the best of our knowledge, this is the first report to document the endeavor for unearthing the chemical repertoire of sesquiterpenoids in *Penicillium* species through overexpressing of exogeneous activator BerA involved in meroterpenoid berkeleyacetals biosynthesis. Filamentous fungi are an important source of terpenoids, but many of the corresponding biosynthetic gene clusters (BGCs) are silent in laboratory conditions. Markedly, as for terpenoids biosynthesis, most encoding BGCs are devoid of regulatory genes which hinder the metabolic engineering via overexpressing endogenous pathway-specific regulators. To investigate the biosynthetic potential of these sesquiterpenoid BGCs, heterologous expression strategies using different host cells have been commonly developed to characterize the target metabolites [13,14,48,49]. Nevertheless, bioinformatically well-defined BGCs of interest and smooth communication of the biosynthetic components or equipment are limitations and key factors of the expression strategy [14]. As confirmed previously, overexpression of regulator BerA in *N*. *glabra* could significantly stimulate the production of berkeleyacetals, which are meroterpenoids partially derived from sesquiterpenoid pathways [4]. Terpenoids are a large family of structurally diverse secondary metabolites, which are generated by the collaboration of pernyltransferases (PTs) and post-modification enzymes [39]. Remarkably, PTs serves as a central player in biosynthesizing terpenoids, as they could convert the linear polyprenyl pyrophosphates to polycyclic scaffolds through carbocation-initiated cascade reactions. We proposed that possible mechanism of this activation strategy is that transcriptional factor BerA could activate the expression of UbiA-type PTs or TCs, exemplified as BerG [4], *Pb*-BisA, and *Pb*_AtsA. Therefore, when employed in metabolic engineering of *P*. *brasilianum*, overexpression of BerA could unlock the down-regulated or cryptic BGCs and initiate biosynthesis of target sesquiterpenoids (Fig. 1, Fig. 2). Results from real-time PCR quantification analysis also supported the up-regulated transcription of two BGCs responsible for arpenibisabolanes and arpenicarotanes biosynthesis in the mutant strain *Pb*-OE:*berA* (Fig. 6B). Previous studies have already demonstrated that endogenous pathway-specific activator-based metabolic engineering could facilitate the up-regulation of target metabolic pathways and assisted for bioprospecting for new SMs [3,4]. Our strategy based on exogenous activator BerA involved in meroterpenoids production could lead to the activation of sesquiterpenoids encoding gene clusters. This further indicates that regulator encoded in meroterpenoid BGCs could be used for sesquiterpenoids exploitation, which broadens the potential application of the STSs-targeted mining strategy.

Although daucane- and bisabolene-type sesquiterpenoids have been isolated from genera *Aspergillus*, *Penicillium*, and *Trichoderma*, respectively [30,35,38,50]. In the present study, interestingly, two classes of structurally different sesquiterpenoids were isolated from *P*. *brasilianum* when the silent or down-regulated encoding BGCs were induced by exogenous activator BerA. Aspterric acid was originally reported as an herbicidal metabolite from the fungus *A*. *terreus* [51]. Recently, daucane terpenoids including penigrisacids (A-D) and piltunines (A-F) were isolated from the marine fungus *P*. *griseofulvum* and *P*. *piltunense*, respectively [35,50]. Moreover, aspenicarotane B was isolated from the coculture system including *P*. *brasilianum* and *Armillaria mellea* in our previous study [33]. This class of compounds is "5 + 7" bicyclic sesquiterpenes exhibiting multiple oxidations, varying ether bond formation (C_2_–C_15_ or C_2_–C_6_), and methylations. Markedly, aspterric acid was reportedly serving as a potent inhibitor of dihydroxyacid dehydratase (IC_50_ = 0.31–0.5), which causes the inhibition of root development and plant growth [38,51]. In addition, as for bisabolene-derived sesquiterpenoids, 12-acetoxybisabolen-1-ol and (*Z*)-12-acetoxybisabol-1-one from *T*. *asperellum* exhibiting inhibitory activities (4–16 μg/mL) against *V*. *harveyi* and *V*. *alginolyticus* were reported recently [30]. This implies that the two classes of sesquiterpenoids might possess herbicidal or antibacterial bioactivities, respectively. However, aspenicarotanes and arpenibisabolanes did not show activity in standard cytotoxic assays when tested against MCF-7, A549, and HepG2 cell lines.

To summarize, our report established an exogeneous regulator-based genetically-modified activation strategy and this strategy would be an efficient complement to other approaches of globally activating silent BGCs for sesquiterpenoid biosynthesis. Through overexpressing heterologous pathway-specific BerA from *N*. *glabra*, the mutant strain significantly increased the production of sesquiterpene-derived metabolites and enabled us to isolate and fully characterize three arpenibisabolane and three arpenicarotane derivatives, respectively. In addition, a hypothetic biosynthetic pathway of isolated metabolites was proposed. These findings will enrich the chemical diversity of sesquiterpenoids from *Penicillium* genus and help to unearth the unexploited sesquiterpenoids using pathway activator BerA of berkeleyacetals biosynthesis. In future work, with the two genetic clusters, downstream biosynthetic studies and metabolic engineering are currently under investigation in our laboratory.

## CRediT authorship contribution statement

**Wenni He:** Visualization, Investigation, Data curation. **Xiaoting Rong:** Methodology, Investigation. **Hui Lv:** Methodology, Data curation. **Lihua Zhang:** Software, Methodology. **Jinglin Bai:** Visualization, Validation. **Lu Wang:** Resources, Methodology. **Liyan Yu:** Writing – review & editing, Conceptualization. **Lixin Zhang:** Writing – review & editing, Supervision, Conceptualization. **Tao Zhang:** Writing – review & editing, Writing – original draft, Supervision, Investigation, Funding acquisition, Conceptualization.

## Availability of data and materials

All data generated during this study are included in this article, and all material is available upon request.

## Consent for publication

All authors approved the publication.

## Funding

This work was financially supported by the 10.13039/501100001809National Natural Science Foundation of China (31872617), the CAMS Innovation Fund for Medical Sciences (CIFMS) (2021-I2M-1-055, 2019-I2M-1-005), and the National Microbial Resource Center (NMRC-2024-3), and the central level, scientific research institutes for basic R & D fund business (3332018097).

## Declaration of competing interest

Lixin Zhang is Editor-in-Chief for Synthetic and Systems Biotechnology and was not involved in the editorial review or the decision to publish this article. Other authors declare that they have no known competing financial interests or personal relationships that could have appeared to influence the work reported in this paper.
