# Supplementary material for: Genetically-modified activation strategy facilitates the discovery of sesquiterpene-derived metabolites from Penicillium brasilianum
Source: Synth Syst Biotechnol. 2024 Dec 25;10(2):391–400. doi: 10.1016/j.synbio.2024.12.006 (PMC11745945; doi:10.1016/j.synbio.2024.12.006)
Supplement: Multimedia component 1 [file mmc1.pdf]

Supplementary information

Genetically-modified activation strategy facilitates the discovery of sesquiterpene-derived metabolites from *Penicillium brasilianum*

Wenni He[†,1], Xiaoting Rong[†,1], Hui Lv[1], Lihua Zhang[2], Jinglin Bai[1], Lu Wang[1], Liyan Yu[1*], Lixin Zhang[3*], Tao Zhang[1*]

[1]Institute of Medicinal Biotechnology, Chinese Academy of Medical Sciences & Peking Union Medical College, Beijing 100050, China

[2]State Key Laboratory of Component-based Chinese Medicine, Institute of Traditional Chinese Medicine, Tianjin University of Traditional Chinese Medicine, Tianjin, 301617, People's Republic of China.

[3]State Key Laboratory of Bioreactor Engineering, East China University of Science & Technology, Shanghai 200237, China

[†]These authors contributed equally to this work.

[*]**Corresponding authors.**

E-mail addresses: zhangt218@hotmail.com (Tao Zhang), yly@cpcc.ac.cn (Liyan Yu), lxzhang@ecust.edu.cn (Lixin Zhang).

Table of content

Identification of compounds **2-3**, and **5-6**.......3

Supplementary tables.......5

Table S1 ^{1}H and ^{13}C NMR Data for compounds **2** and **5** in CD_3OD.......5

Table S2 Cytotoxicity of metabolites (**1-6**) and cisplatin against three cell lines including MCF-7, A549, and HepG2.......6

Table S3 Antibacterial activities of isolated compounds **1**–**6** (MIC, µg/mL).......6

Supplementary figures.......7

Figure S1. Diagnostic PCR characterization of mutants including *Pb*-OE::*berA*-R3 and *Pb*-OE::*berA*-R7.......7

Figure S2. Key NOESY and HMBC correlations of compound **2** and **5**.......8

Figure S3. Experimental ECD spectrum of compound **4.**.......8

Figure S4. HR ESIMS spectrum of compound **1**.......9

Figure S5. UV spectrum of compound **1**.......9

Figure S6. ^{1}H NMR (600 MHz, CD_3OD) spectrum of compound **1**.......10

Figure S7. ^{13}C NMR (150 MHz, CD_3OD) spectrum of compound **1**.......10

Figure S8. ^{1}H-^{1}H COSY (600 MHz, CD_3OD) spectrum of compound **1**.......11

Figure S9. HSQC (150 MHz, CD_3OD) spectrum of compound **1**.......11

Figure S10. HMBC (150 MHz, CD_3OD) spectrum of compound **1**.......12

Figure S11. NOESY (600 MHz, CD_3OD) spectrum of compound **1**.......12

Figure S12. HR ESIMS spectrum of compound **4**.......13

Figure S13. UV spectrum of compound **4**.......13

Figure S14. ^{1}H NMR (600 MHz, CD_3OD) spectrum of compound **4**.......14

Figure S15. ^{13}C NMR (150 MHz, CD_3OD) spectrum of compound **4**.......14

Figure S16. ^{1}H-^{1}H COSY (600 MHz, CD_3OD) spectrum of compound **4**.......15

Figure S17. HSQC (150 MHz, CD_3OD) spectrum of compound **4**.......15

Figure S18. HMBC (150 MHz, CD_3OD) spectrum of compound **4**.......16

Figure S19. NOESY (600 MHz, CD_3OD) spectrum of compound **4.**.......16

Figure S20. HR ESIMS spectrum of compound 2.......17

Figure S21. ^{1}H NMR (600 MHz, CD_3OD) spectrum of compound **2**.......17

Figure S22. ^{13}C NMR (150 MHz, CD_3OD) spectrum of compound **2**.......18

Figure S23. ^{1}H-^{1}H COSY (600 MHz, CD_3OD) spectrum of compound **2**.......18

Figure S24. HSQC (150 MHz, CD_3OD) spectrum of compound 2.......19

Figure S25. HMBC (150 MHz, CD_3OD) spectrum of compound **2**.......19

Figure S26. NOESY (600 MHz, CD_3OD) spectrum of compound **2**.......20

Figure S27. ^{1}H NMR (600 MHz, CD_3OD) spectrum of compound **3**.......20

Figure S28. ^{13}C NMR (150 MHz, CD_3OD) spectrum of compound **3**.......21

Figure S29. HR ESIMS spectrum of compound **5**.......21

Figure S30. ^{1}H NMR (600 MHz, CD_3OD) spectrum of compound **5**.......22

Figure S31. ^{13}C NMR (150 MHz, CD_3OD) spectrum of compound **5**.......22

Figure S32. ^{1}H-^{1}H COSY (600 MHz, CD_3OD) spectrum of compound **5**.......23

Figure S33. HSQC (150 MHz, CD_3OD) spectrum of compound 5.......24

Figure S34. HMBC (150 MHz, CD_3OD) spectrum of compound **5**.......24

Figure S35. NOESY (600 MHz, CD_3OD) spectrum of compound **5.**.......25

Figure S36. ^{1}H NMR (600 MHz, $CDCl_3$) spectrum of compound **6**.......25

Figure S37. ^{13}C NMR (150 MHz, $CDCl_3$) spectrum of compound **6.** 26

Identification of compounds **2-3**, and **5-6**

Compound **2** was purified as colorless oil, and its molecular formula was established as $C_{15}H_{20}O_4$ based on HRESIMS data, [*m*/*z* 265.14264 $[M+H]^+$ (calcd for $C_{15}H_{21}O_4$ 265.14344)] corresponding to six degrees of unsaturation. The ^1H and ^{13}C NMR data (Table S1, Supplementary Information) were found to be accordant with those of **1**. Further analysis of 2D NMR spectroscopic data, including HSQC, HMBC, and ^1H-^1H COSY verified that compounds **1** and **2** shared identical planar structures. However, the NOE spectrum showed correlations between H_3-14 (δ_H 1.93) / H-5 (δ_H 3.02) and H_3-13 (δ_H 1.81) / H-9 (δ_H 2.37) which determined that the configurations of double bonds $\Delta^{6,7}$ and $\Delta^{10,11}$ were assigned as Z and E respectively (Fig. S2). The absolute configuration of C-4 was determined as 4*S* by ECD calculation (Fig. 4). On the grounds of ^1H and ^{13}C NMR data, along with the analysis of data with our previous co-culture study [1], compound **2** was elucidated and suggested as arpenibisabolane A (Fig. 1).

Compound **3** was purified as yellow oil. Based on the analysis of ^1H and ^{13}C NMR data, along with the investigation of literature data [3], compound **3** was elucidated and suggested as eupenicisirenins A (Figure 1). The NMR data were shown as follows, in ^1H NMR data (in CD_3OD, 600 MHz): δ_H 7.20 (1H, dd, *J* =5.5, 3.0 Hz, H-2), 5.14 (1H, m, H-10), 4.18 (1H, m, H-5), 2.85 (1H, dd, *J* = 17.5, 8.5 Hz, H-4), 2.15 (2H, m, H-9), 1.75 (1H, m, H-4), 1.68 (3H, s, H-12), 1.63 (3H, s, H-13), 1.62 (3H, m, H-1), 1.45 (2H, m, H-6 and H-8), 1.28 (1H, m, H-8), and 1.00 (3H, s, H-14). The ^{13}C NMR data (in CD_3OD, 151MHz): δ_C 170.1 (C-15), 140.4 (C-2), 132.3 (C-11), 127.0 (C-3), 125.3 (C-10), 66.5 (C-5), 45.0 (C-8), 35.4 (C-7), 33.4 (C-6), 33.1 (C-4), 28.7 (C-1), 26.5 (C-12), 25.9 (C-94), 17.7 (C-13), and 13.6 (C-14).

Compound **5** was deduced to possess the molecular formula of $C_{15}H_{22}O_4$ by analyzing HRESIMS data at *m*/*z* 267.15802 $[M+H]^+$ (calcd for $C_{15}H_{23}O_4$ 267.15909), corresponding to five degrees of unsaturation. Upon comparison of the 1D NMR data (Table S1, Supplementary Information) and HR-ESIMS data of **4** and **5**, they shared the same carotane sesquiterpene skeleton except for the disappearance of an acetyl group in **5**. It was further confirmed by the HMBC correlations (Fig. S2) of H-3 (δ_H 3.72) with C-14 (δ_C 176.4), and H-5 (δ_H 2.33 and 1.77) with C-14 (δ_C 176.4). On the

basis of ^{1}H and ^{13}C NMR data, along with the investigation of data with our previous coculture study (personal communication), compound **5** was suggested as arpenicarotane B (Fig. 1).

Compound **6** was purified as yellow oil. Owing to ^{1}H and ^{13}C NMR data, along with the investigation of literature data [5], compound **6** was suggested as aspterric acid (Fig. 1). The NMR data were shown as follows, in ^{1}H NMR data (in $CDCl_3$, 600 MHz): δ_H 4.30 (1H, d, J = 8.8 Hz, H-3), 3.94 (1H, d, J = 8.0 Hz, H-15), 3.50 (1H, d, J = 8.0 Hz, H-15), 2.42 (1H, m, H-10), 2.30-2.40 (3H, m, H-5, H-7, H-10), 2.29 (1H, m, H-2), 2.18 (1H, m, H-6), 2.15 (1H, m, H-2), 2.04 (1H, m, H-5), 1.76 (1H, m, H-6), 1.72 (1H, m, H-9), 1.71 (3H, s, H-12), 1.61 (3H, s, H-13), and 1.52 (1H, m, H-9). The ^{13}C NMR data (in $CDCl_3$, 151MHz): δ_C 178.3 (C-14), 134.5 (C-8), 125.4 (C-11), 82.9 (C-3), 76.4 (C-15), 75.5 (C-4), 55.5 (C-7), 53.1(C-1), 37.0 (C-6), 36.3 (C-2), 33.9 (C-9), 32.3 (C-5), 23.7 (C-10), 23.5 (C-13), and 21.0 (C-12).

References

[1] Rong X, Zhang L, He W, Guo Z, Lv H, Bai J, Yu L, Zhang L, Zhang T. Exploration of diverse secondary metabolites from *Penicillium brasilianum* by co-culturing with *Armillaria mellea*. Applied Microbiology and Biotechnology, 2024, 108(1):462.

Supplementary tables

Table S1 ^{1}H and ^{13}C NMR Data for compounds **2** and **5** in CD_3OD.

	**2**		**5**	
Pos.	δ_C	δ_H	δ_C	δ_H
1	192.7		44.4	
2	129.5	5.83 (1H, m)	47.4	2.08 (1H, dd, *J*=4.8, 13.2), 1.77 (1H, m)
3	164.3		77.5	3.72 (1H, dd, *J*=4.8, 11.4)
4	70.0	4.24 (1H, m)	79.3	
5	38.8	3.02 (1H, m), 2.59 (1H, m)	33.8	2.23 (1H, m), 1.77 (1H, m)
6	129.3		22.3	2.30 (1H, m,), 1.29 (1H, m)
7	149.2		51.2	2.78 (1H, m)
8	35.4	2.53 (1H, m), 2.63 (1H, m)	149.2	
9	28.8	2.37 (2H, m)	126.1	5.57 (1H, t, *J* = 3.0 Hz)
10	143.1	6.78 (1H, td, *J*= 7.9, 1.2 Hz)	49.4	2.16 (1H, m), 2.03 (1H, m)
11	129.3		141.9	
12	171.6		111.9	4.83 (1H, m), 4.73 (1H, m)
13	12.4	1.81 (3H, s)	22.5	1.83 (3H, s)
14	21.0	1.93 (3H, s)	176.4	
15	20.2	2.01 (3H, s)	21.0	1.06 (3H, s)

Table S2 Antibacterial activities of isolated compounds **1**–**6** (MIC, μg/mL). [a]

Comp.	*V. algivorus*	*V. owensii*
**1**	64	128
**2**	64	128
**3**	n.a.	n.a.
**4**	n.a.	n.a.
**5**	n.a.	n.a.
**6**	n.a.	n.a.
chloramphenicol	4	4

[a] n.a.: no activity, MIC >128 μg/mL.

Table S3 Cytotoxicity of metabolites (**1**-**6**) and cisplatin against three cell lines including MCF-7, A549, and HepG2.

Comp.	MCF-7 (μM)	A549 (μM)	HepG2 (μM)
**1**	>100	>100	>100
**2**	>100	>100	>100
**3**	>100	>100	>100
**4**	>100	>100	>100
**5**	>100	>100	>100
**6**	>100	>100	>100
Cisplatin	4.67	4.53	6.24

Supplementary figures

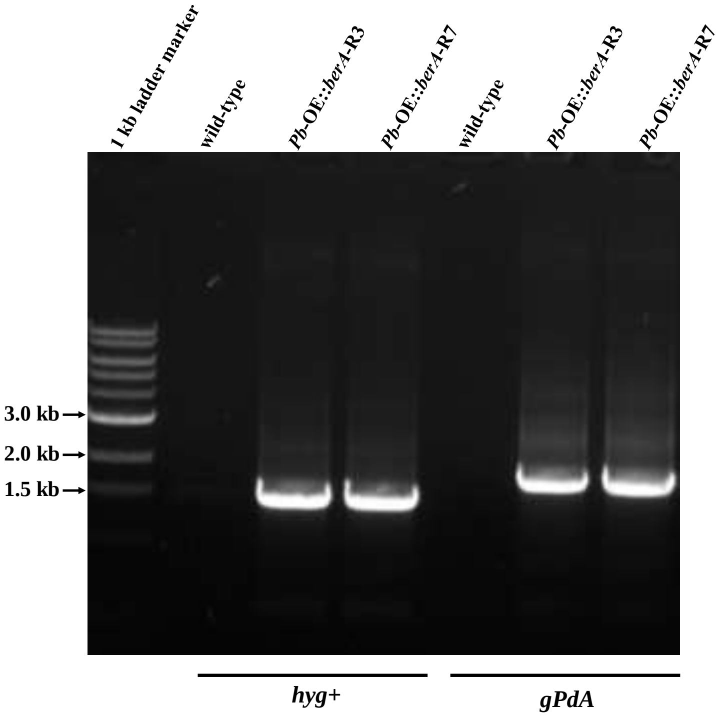

Figure S1. Diagnostic PCR characterization of mutants including *Pb*-OE::*berA*-R3 and *Pb*-OE::*berA*-R7.

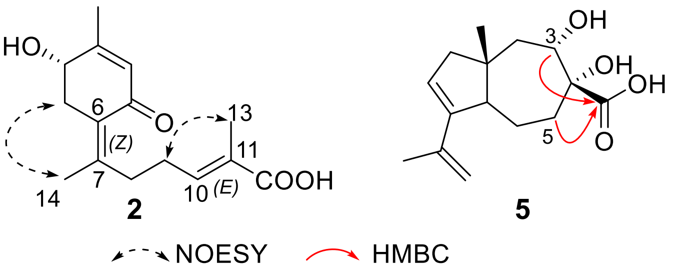

Figure S2. Key NOESY and HMBC correlations of compound **2** and **5**.

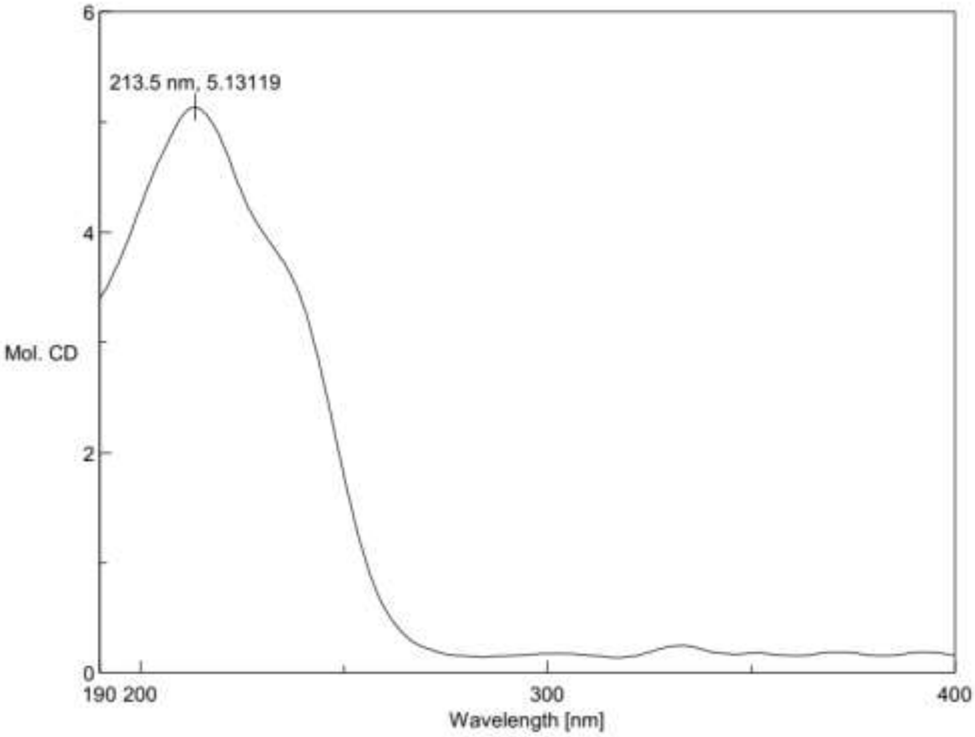

Figure S3. Experimental ECD spectrum of compound **4.**

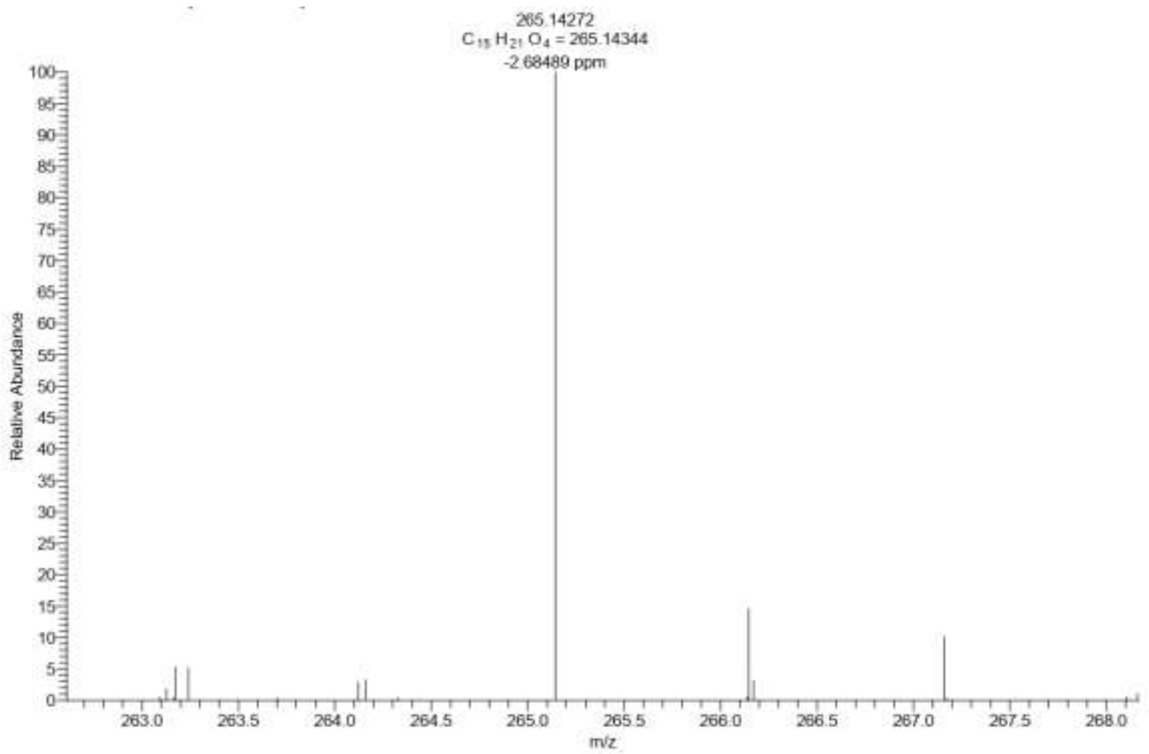

Figure S4. HR ESIMS spectrum of compound **1**.

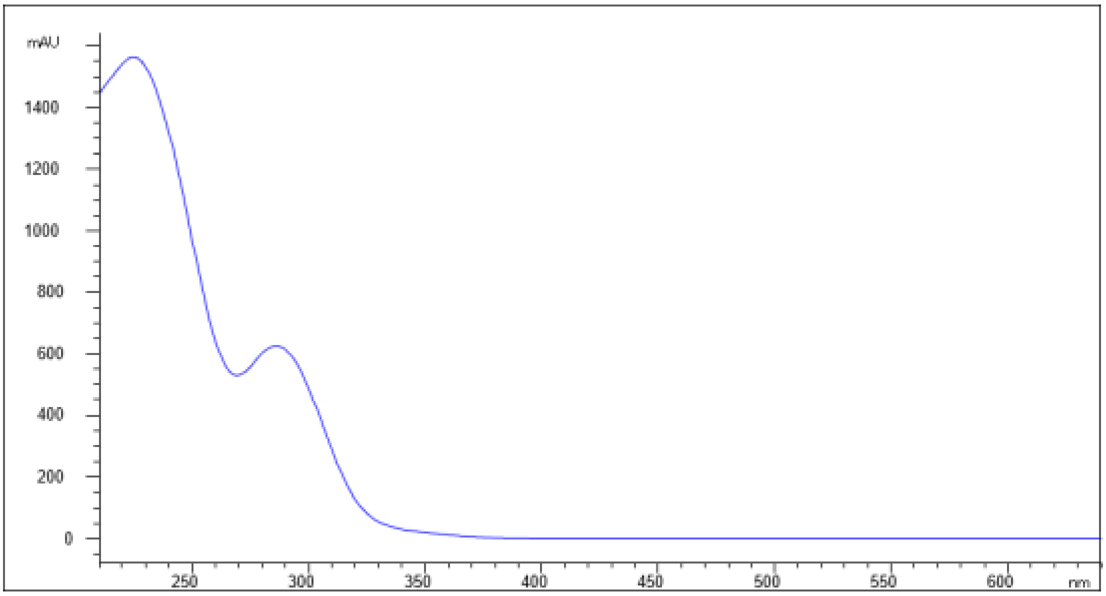

Figure S5. UV spectrum of compound **1**.

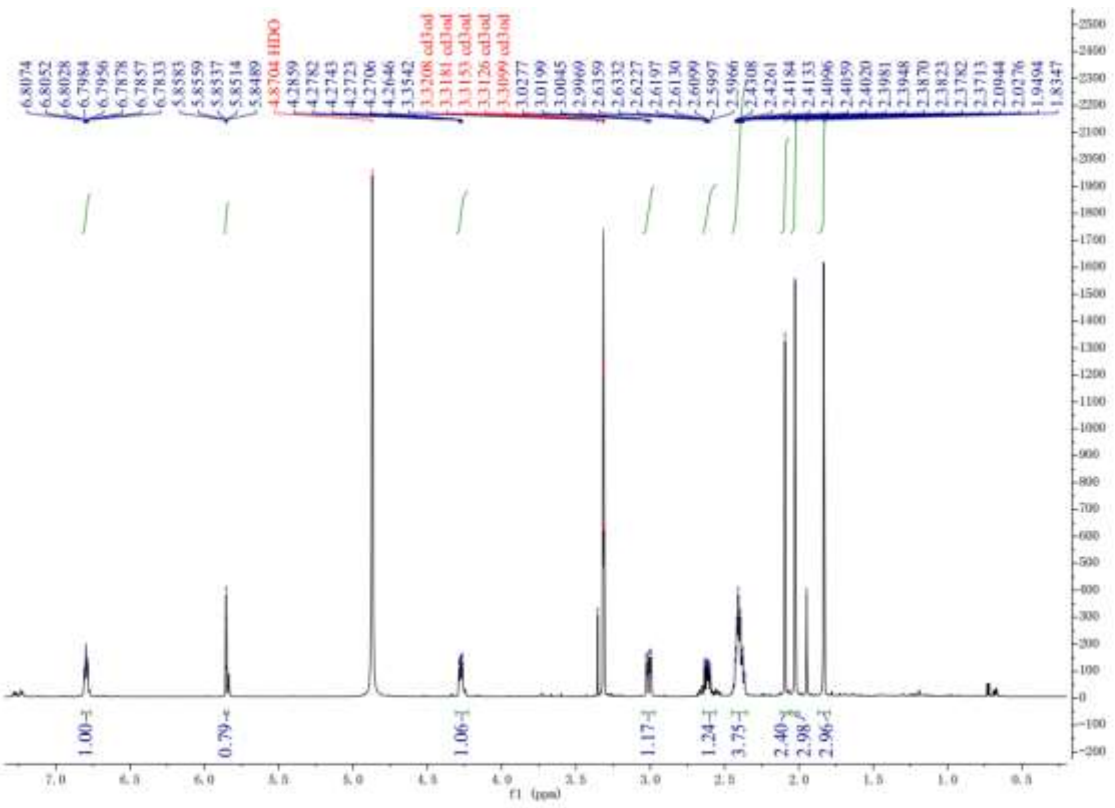

Figure S6. ^{1}H NMR (600 MHz, CD$_3$OD) spectrum of compound **1**.

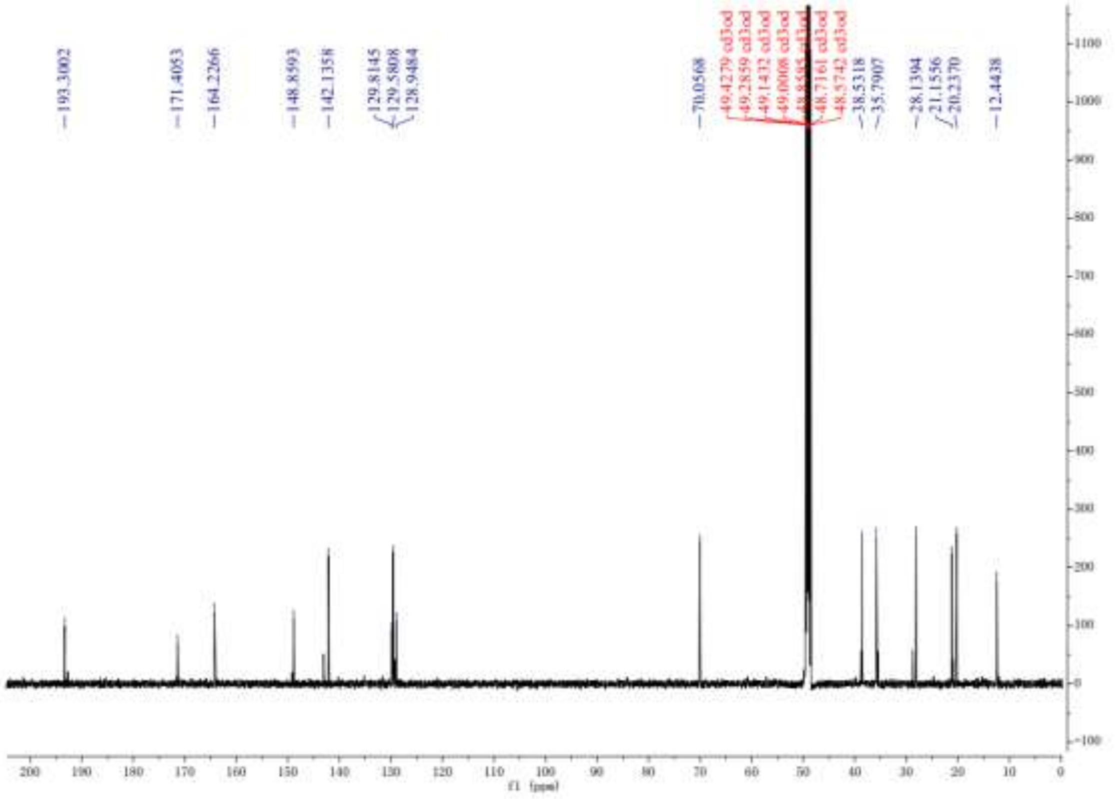

Figure S7. ^{13}C NMR (150 MHz, CD$_3$OD) spectrum of compound **1**.

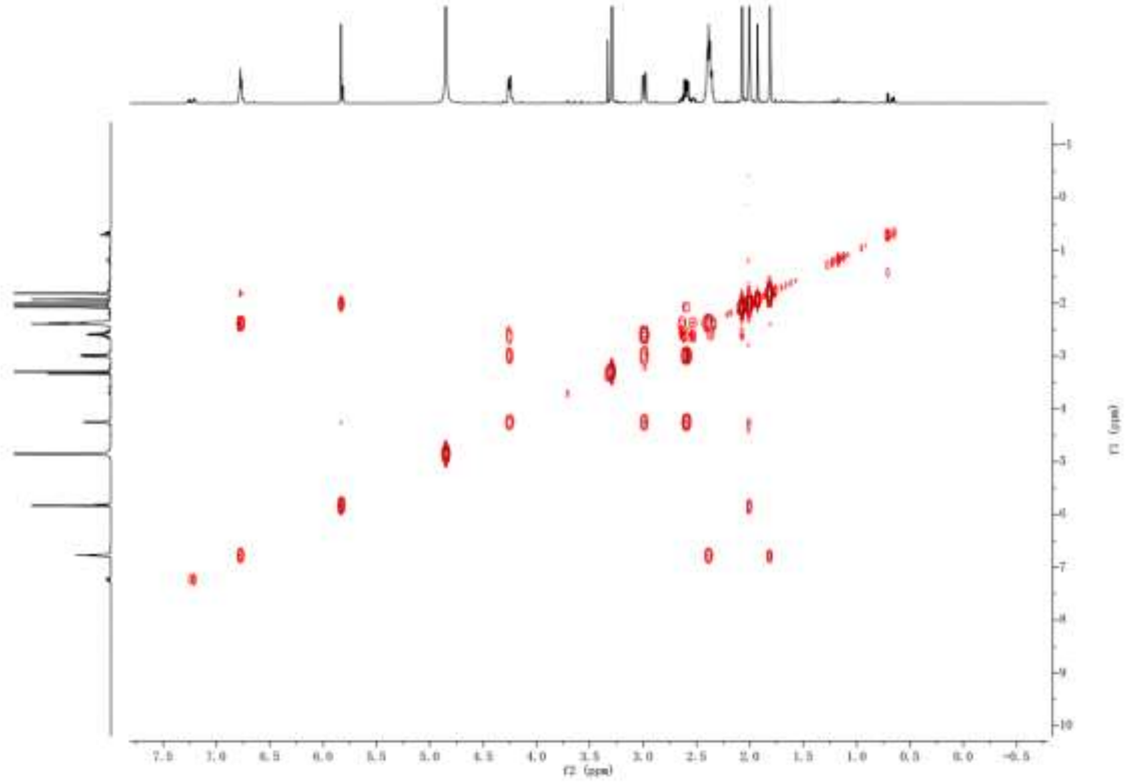

Figure S8. ^{1}H-^{1}H COSY (600 MHz, CD_3OD) spectrum of compound **1**.

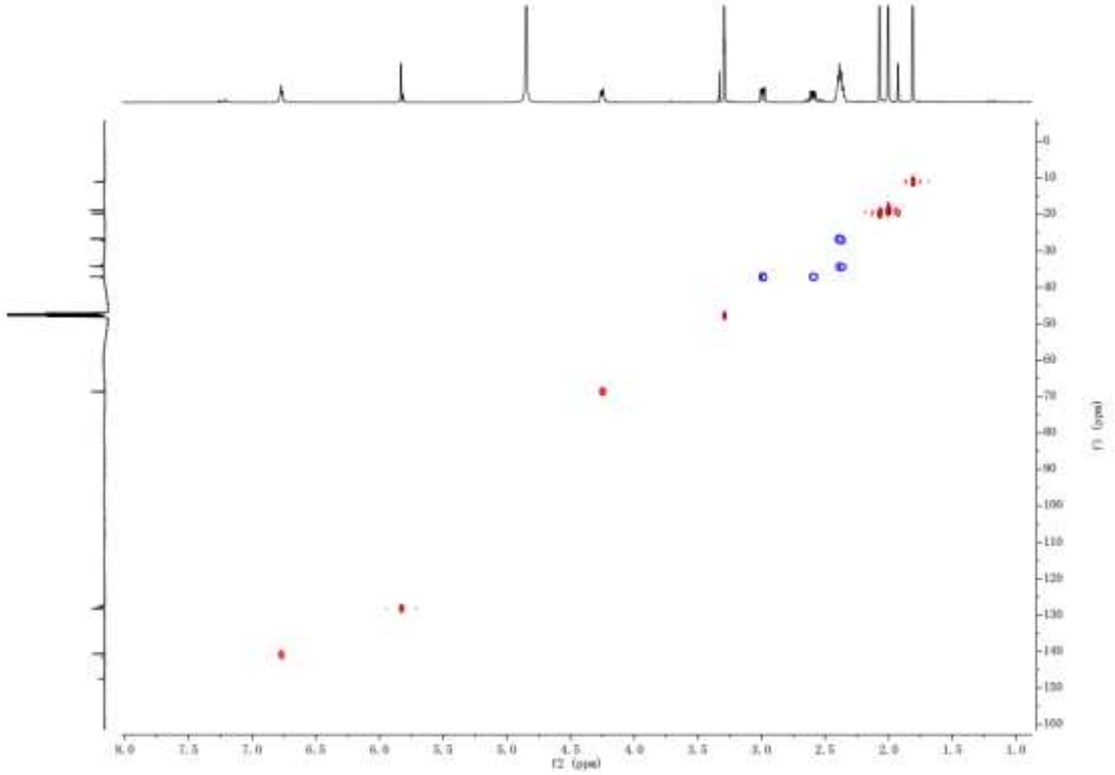

Figure S9. HSQC (150 MHz, CD_3OD) spectrum of compound **1**.

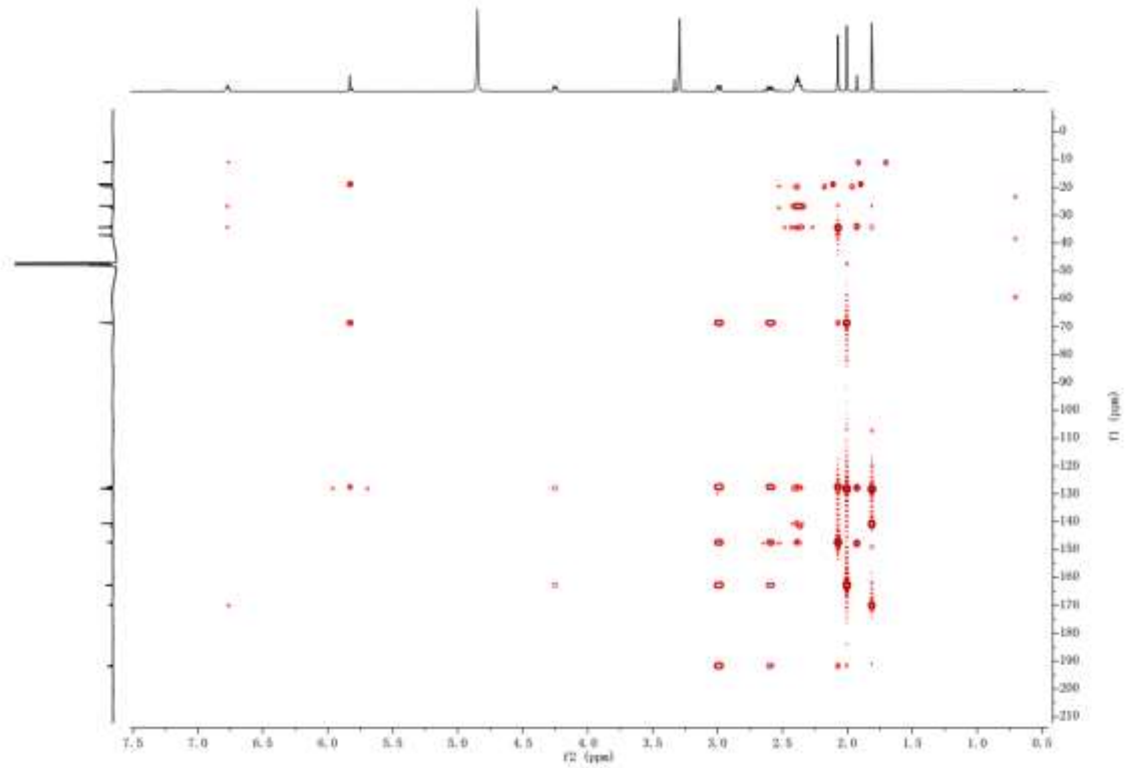

Figure S10. HMBC (150 MHz, CD_3OD) spectrum of compound **1**.

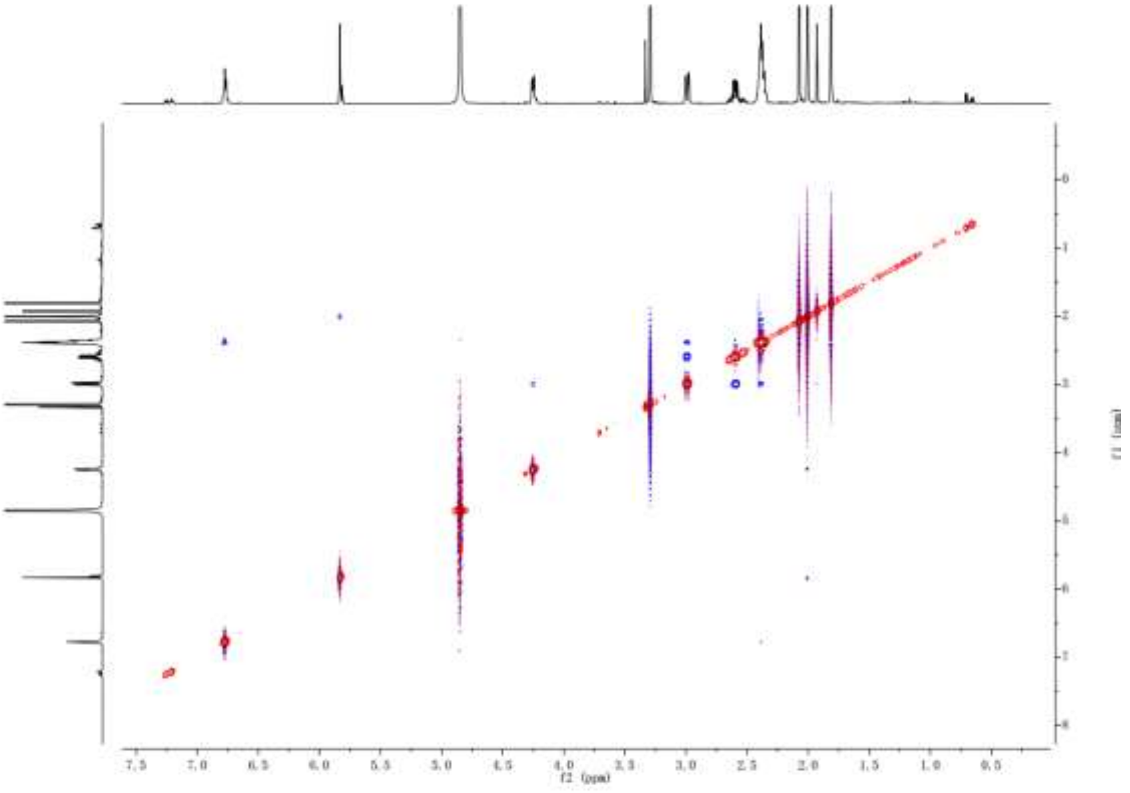

Figure S11. NOESY (600 MHz, CD_3OD) spectrum of compound **1**.

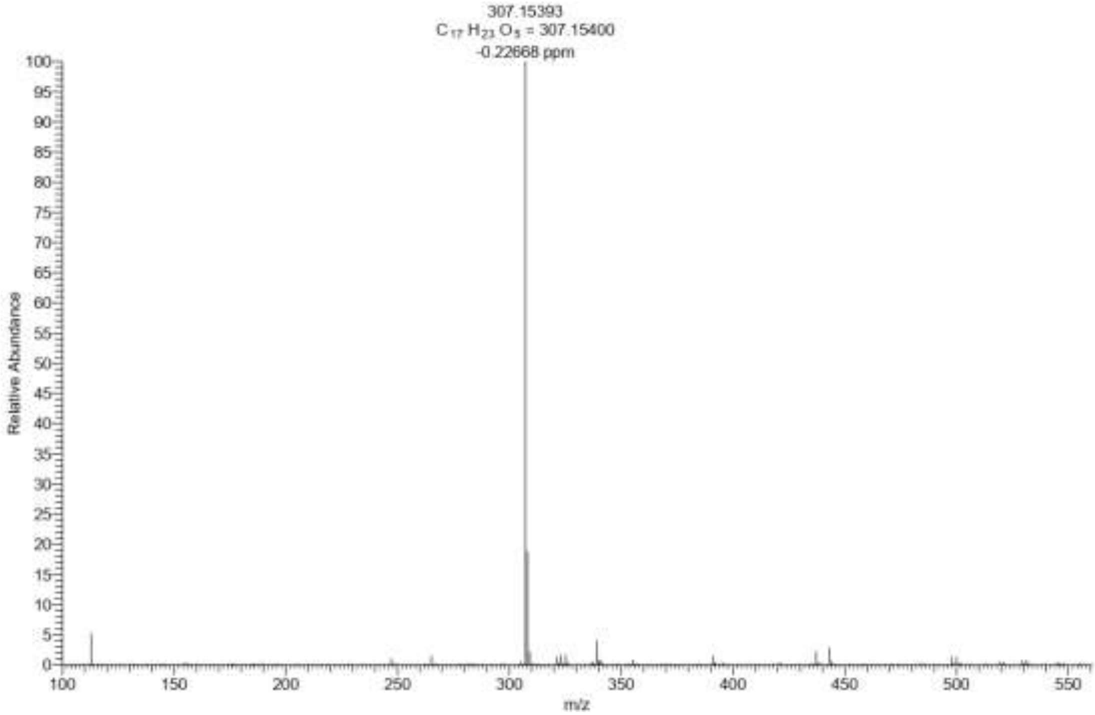

Figure S12. HR ESIMS spectrum of compound **4**.

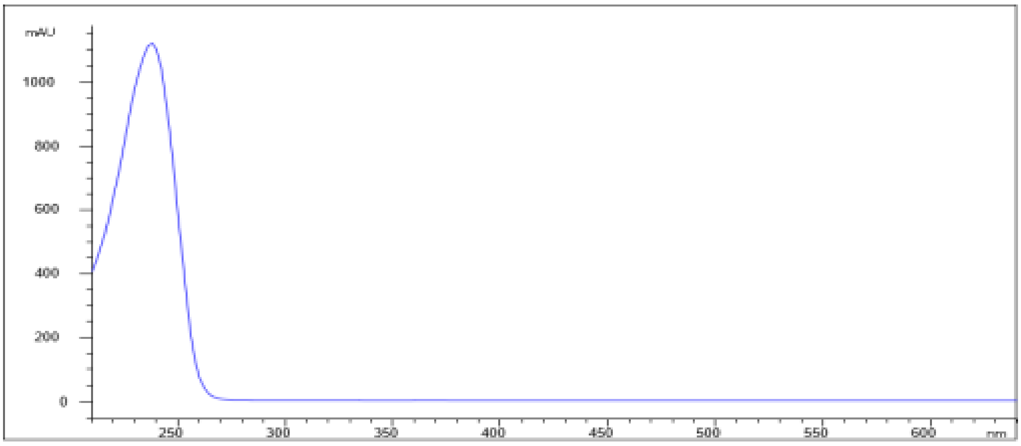

Figure S13. UV spectrum of compound **4**.

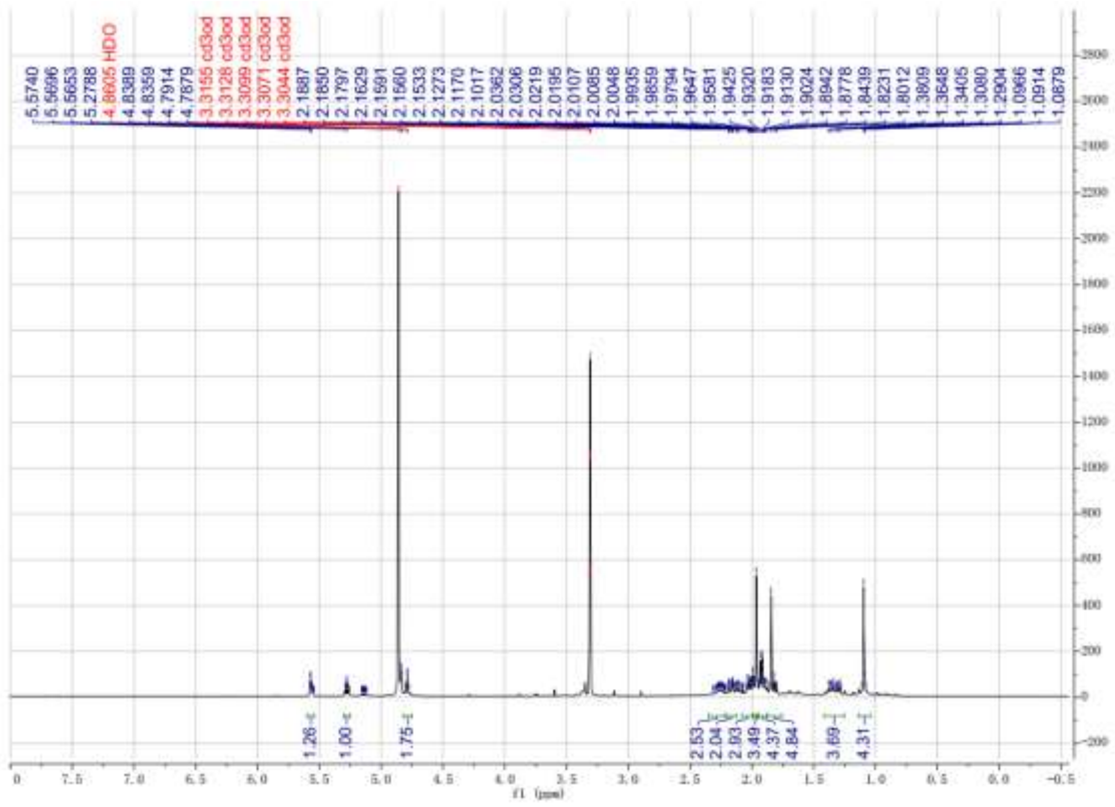

Figure S14. ^{1}H NMR (600 MHz, CD_3OD) spectrum of compound **4**.

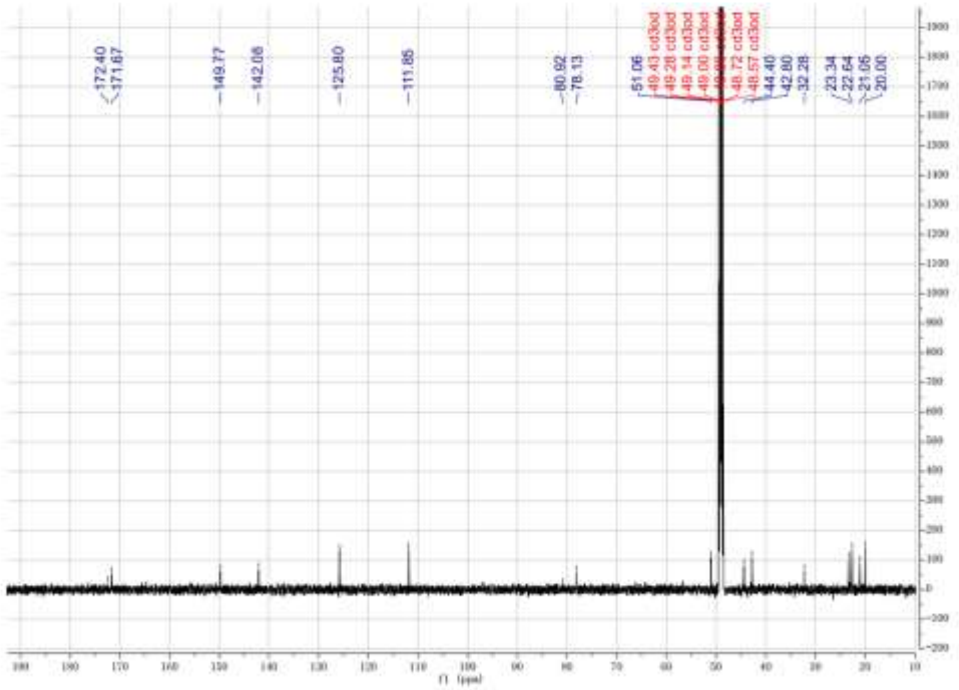

Figure S15. ^{13}C NMR (150 MHz, CD_3OD) spectrum of compound **4**.

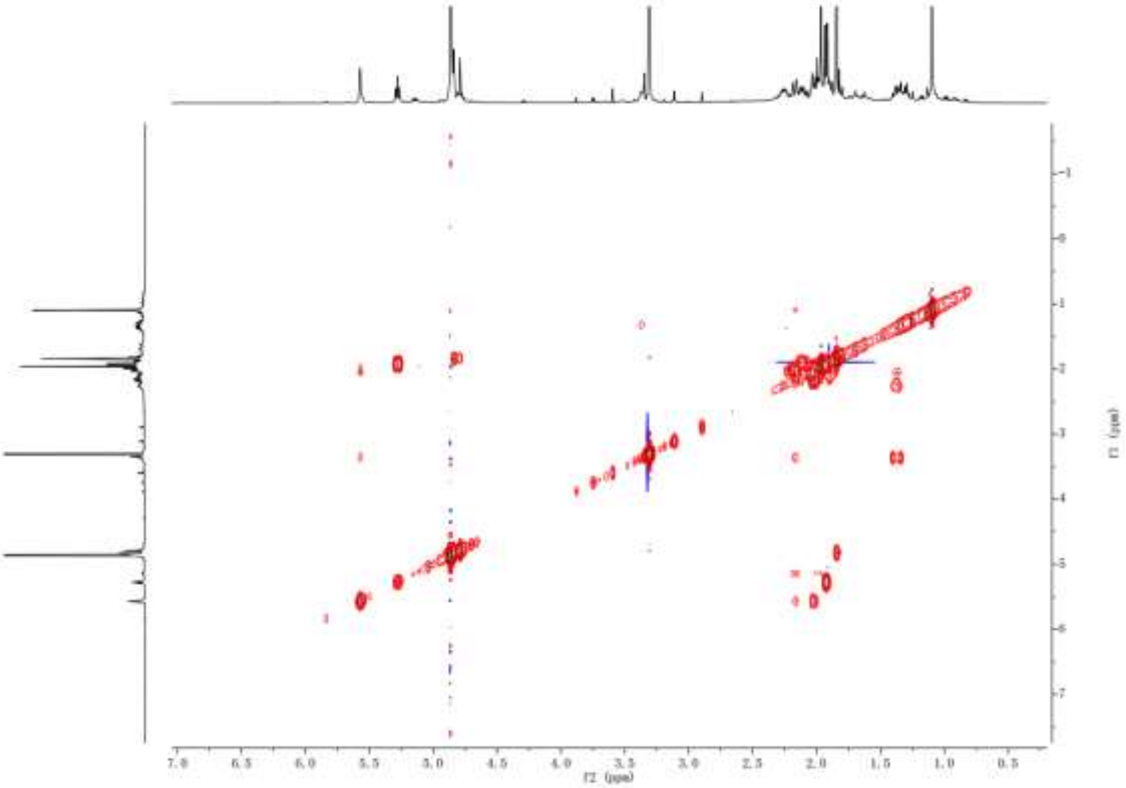

Figure S16. ^{1}H-^{1}H COSY (600 MHz, CD_3OD) spectrum of compound **4**.

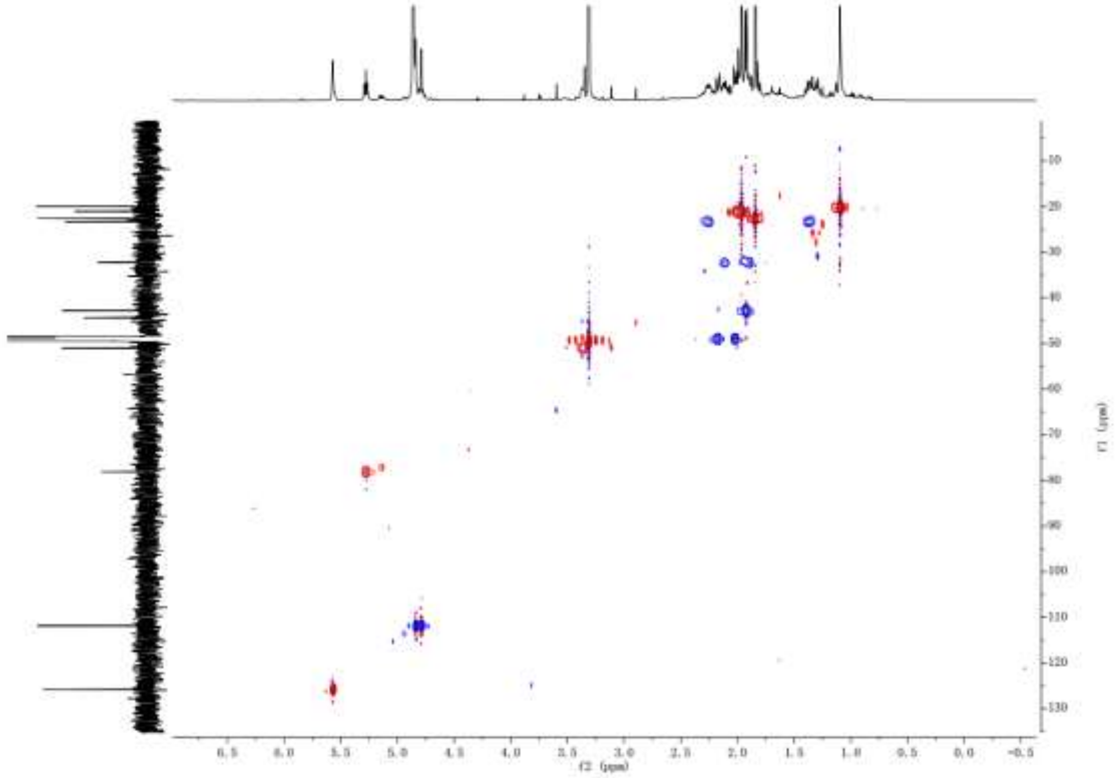

Figure S17. HSQC (150 MHz, CD_3OD) spectrum of compound **4**.

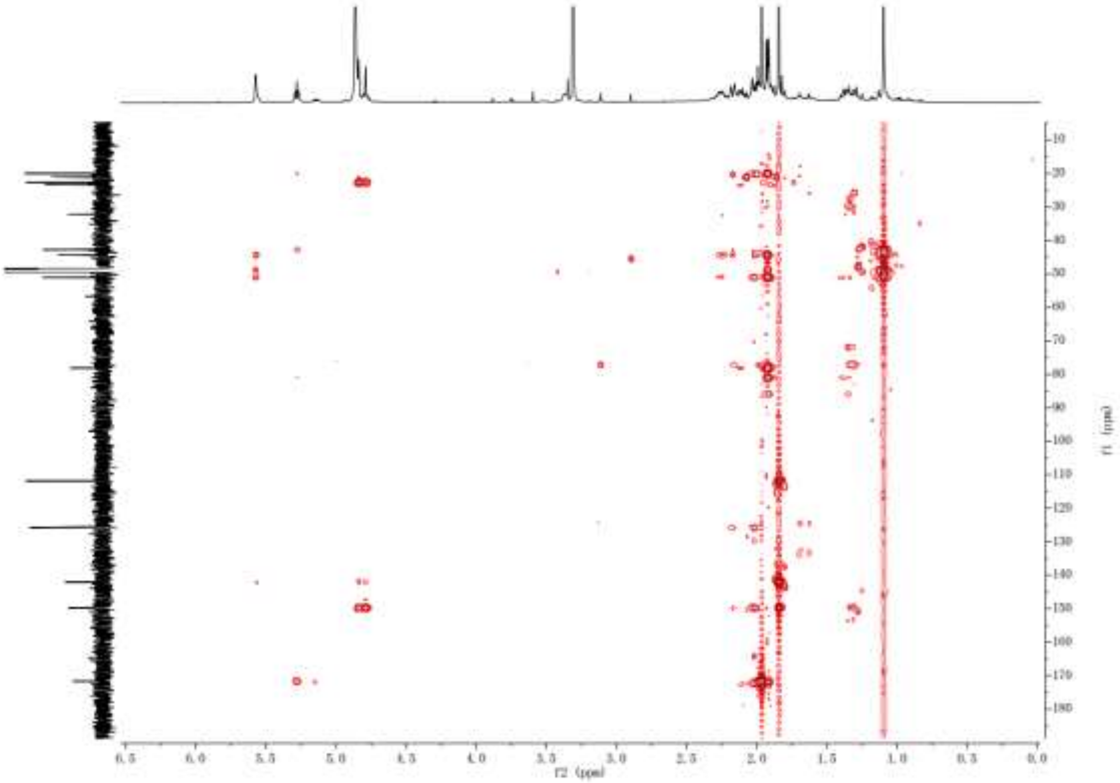

Figure S18. HMBC (150 MHz, CD_3OD) spectrum of compound **4**.

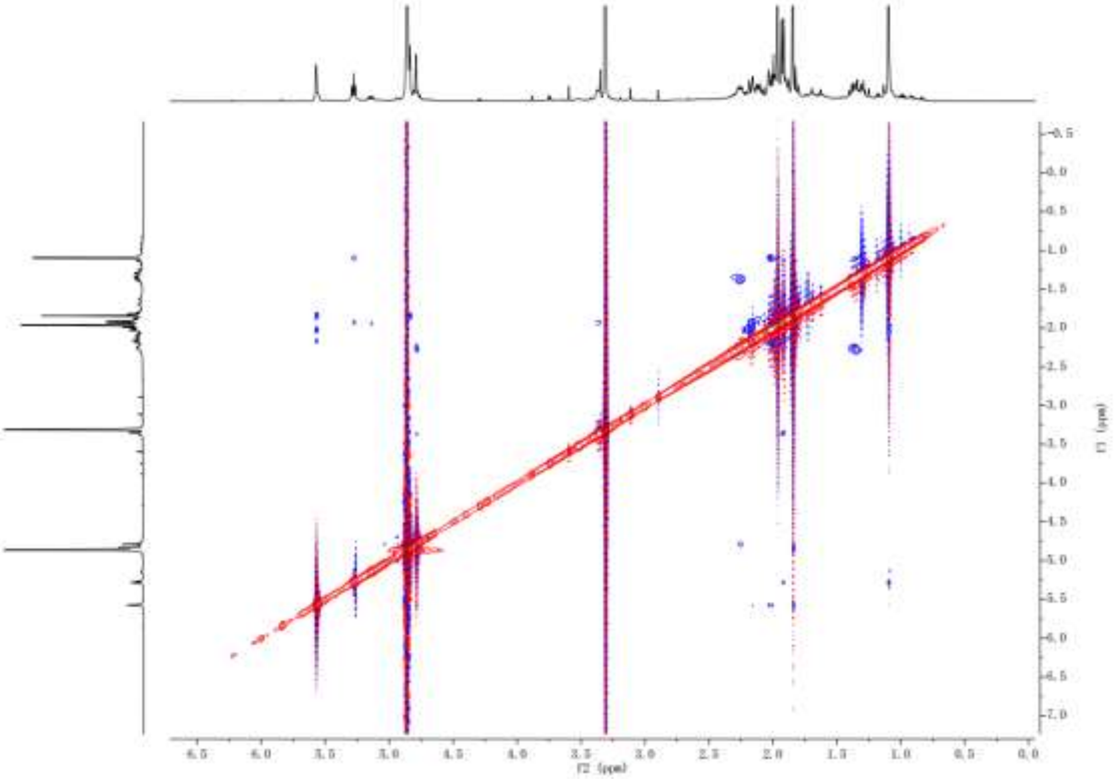

Figure S19. NOESY (600 MHz, CD_3OD) spectrum of compound **4.**

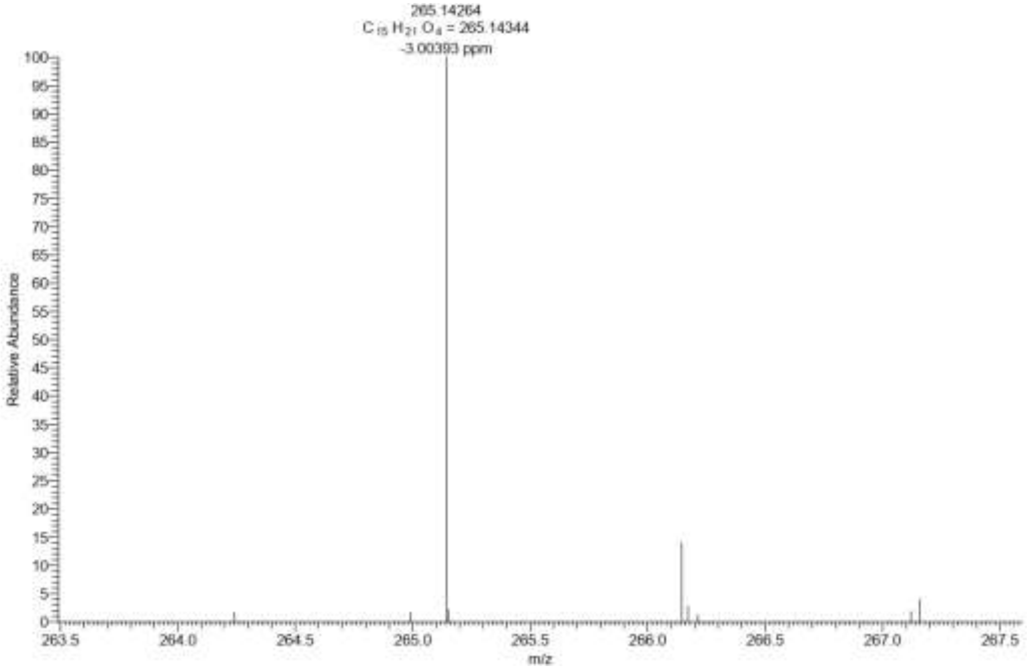

Figure S20. HR ESIMS spectrum of compound **2.**

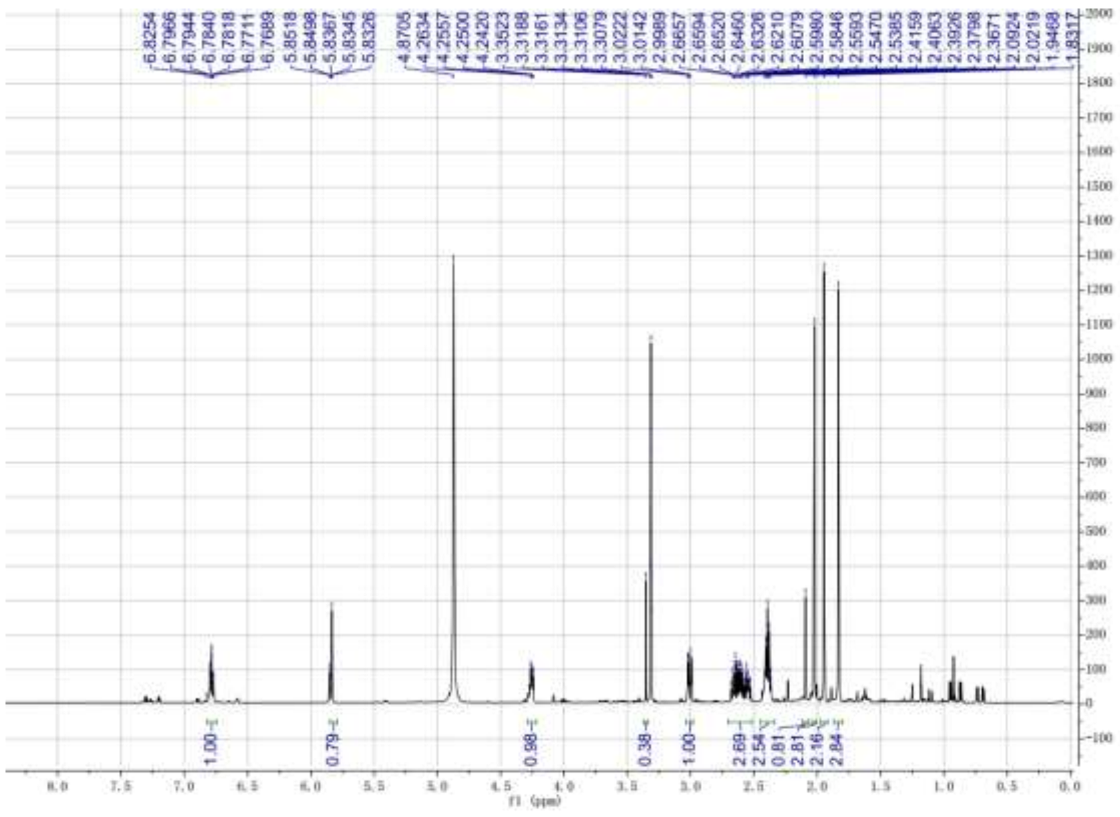

Figure S21. ^{1}H NMR (600 MHz, CD_3OD) spectrum of compound **2**.

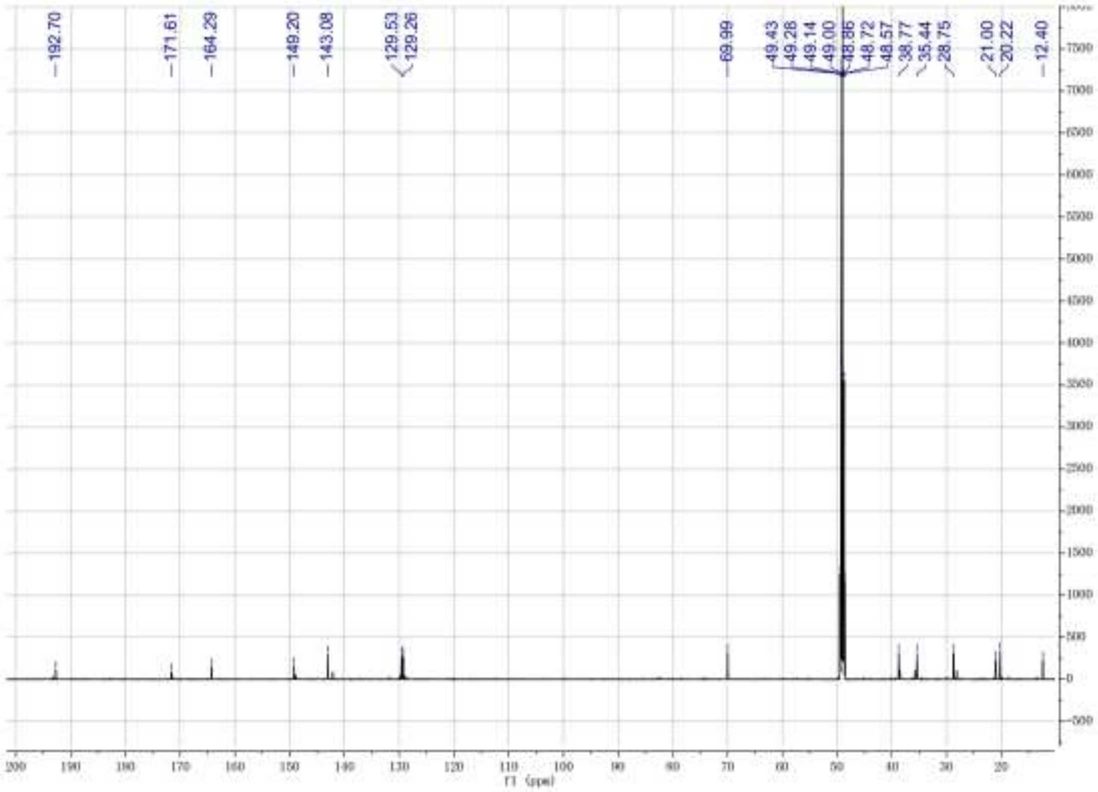

Figure S22. ^{13}C NMR (150 MHz, CD_3OD) spectrum of compound **2**.

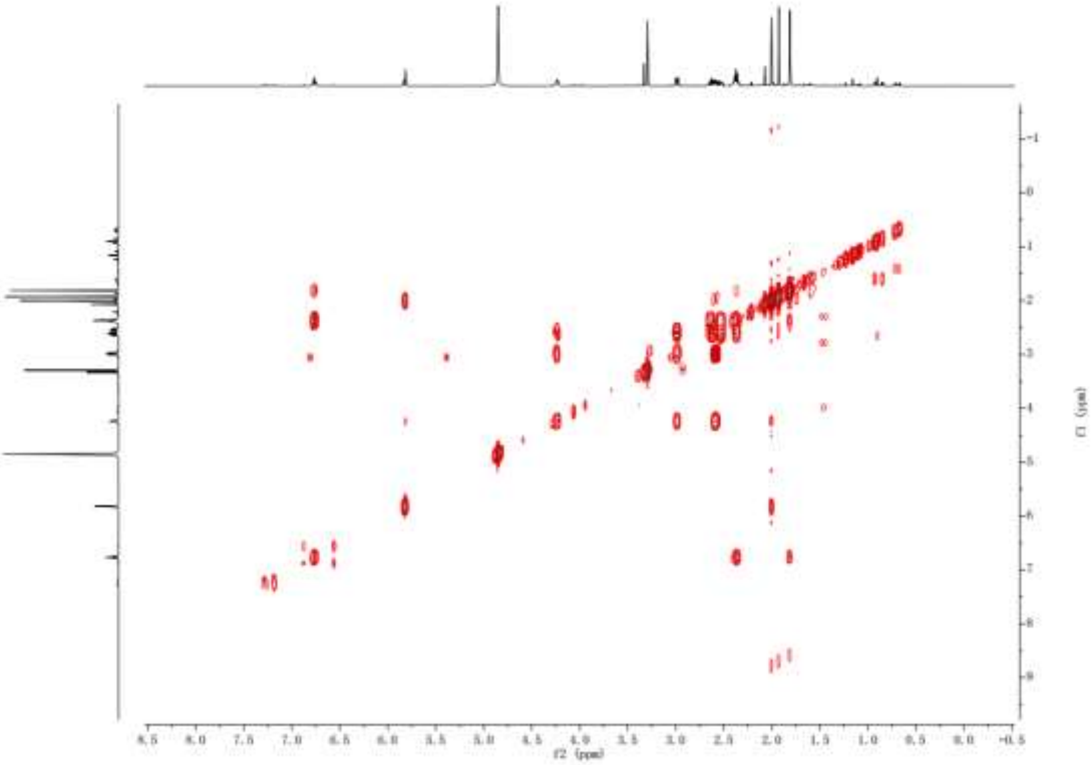

Figure S23. ^{1}H-^{1}H COSY (600 MHz, CD_3OD) spectrum of compound **2**.

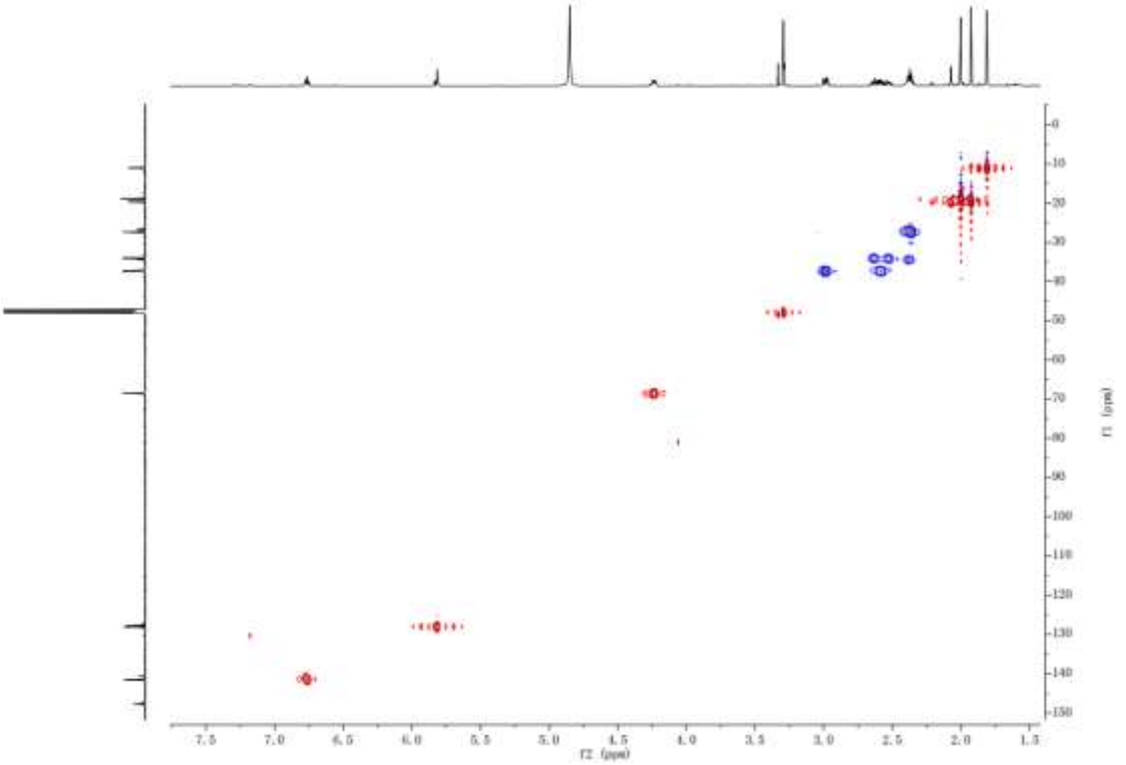

Figure S24. HSQC (150 MHz, CD_3OD) spectrum of compound **2.**

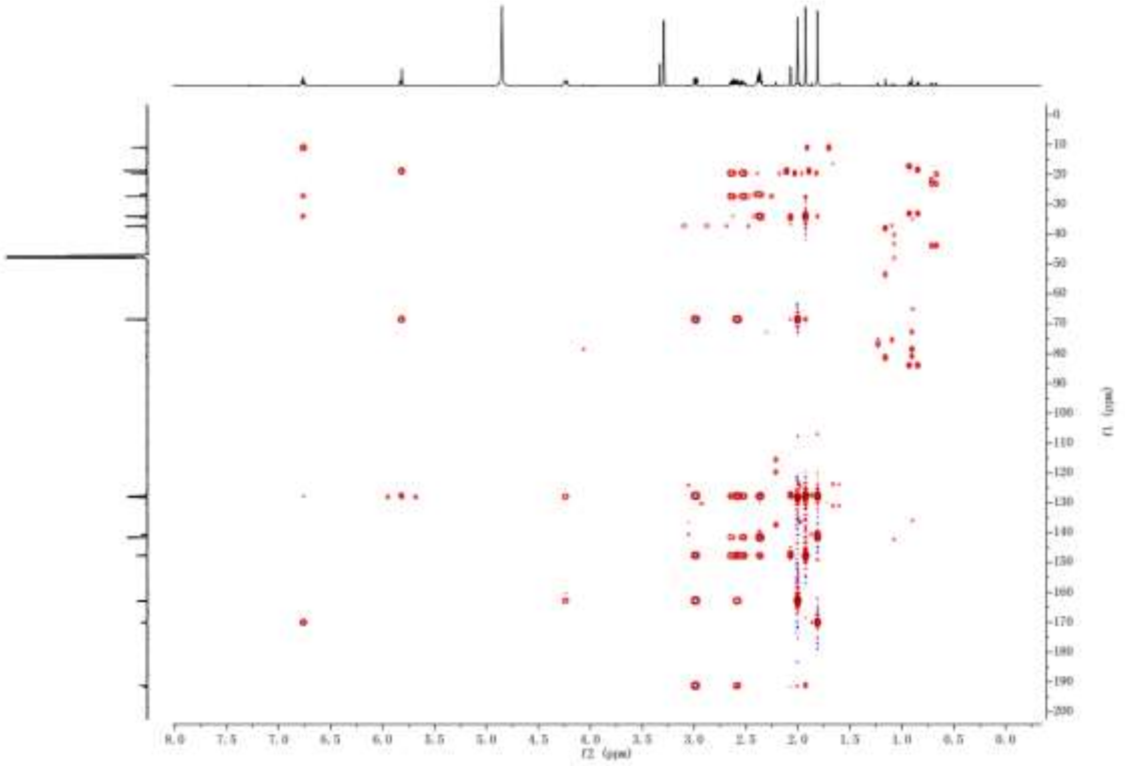

Figure S25. HMBC (150 MHz, CD_3OD) spectrum of compound **2**.

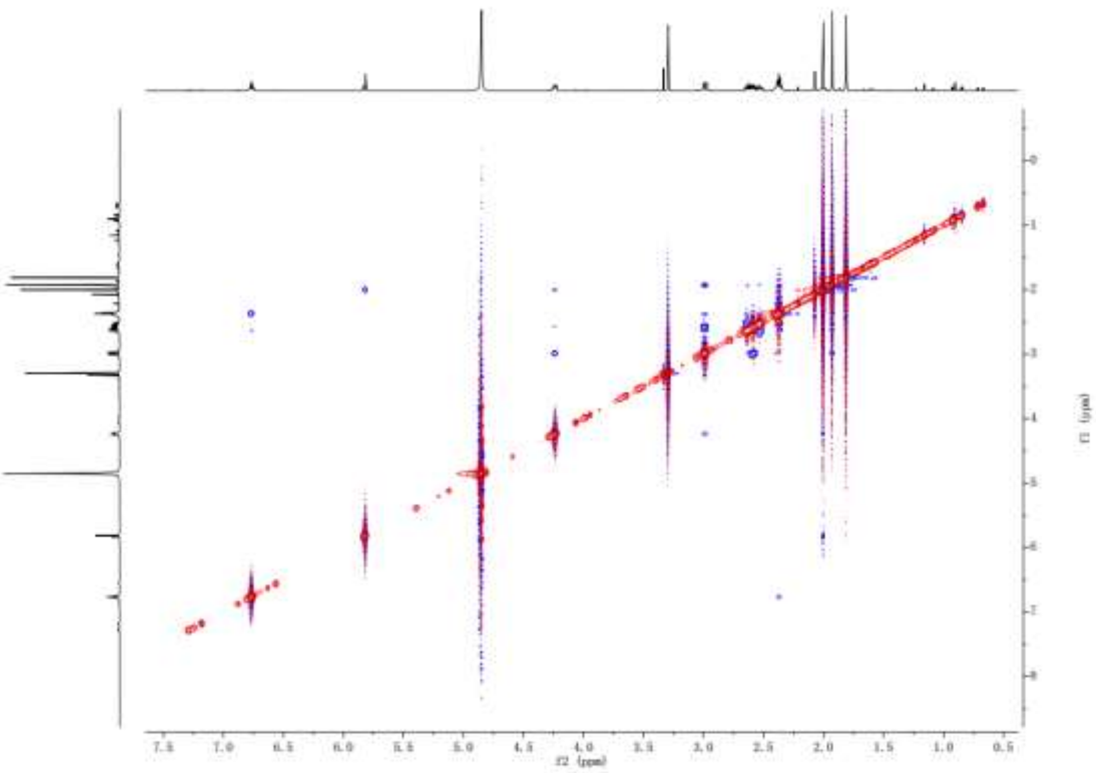

Figure S26. NOESY (600 MHz, CD_3OD) spectrum of compound **2**.

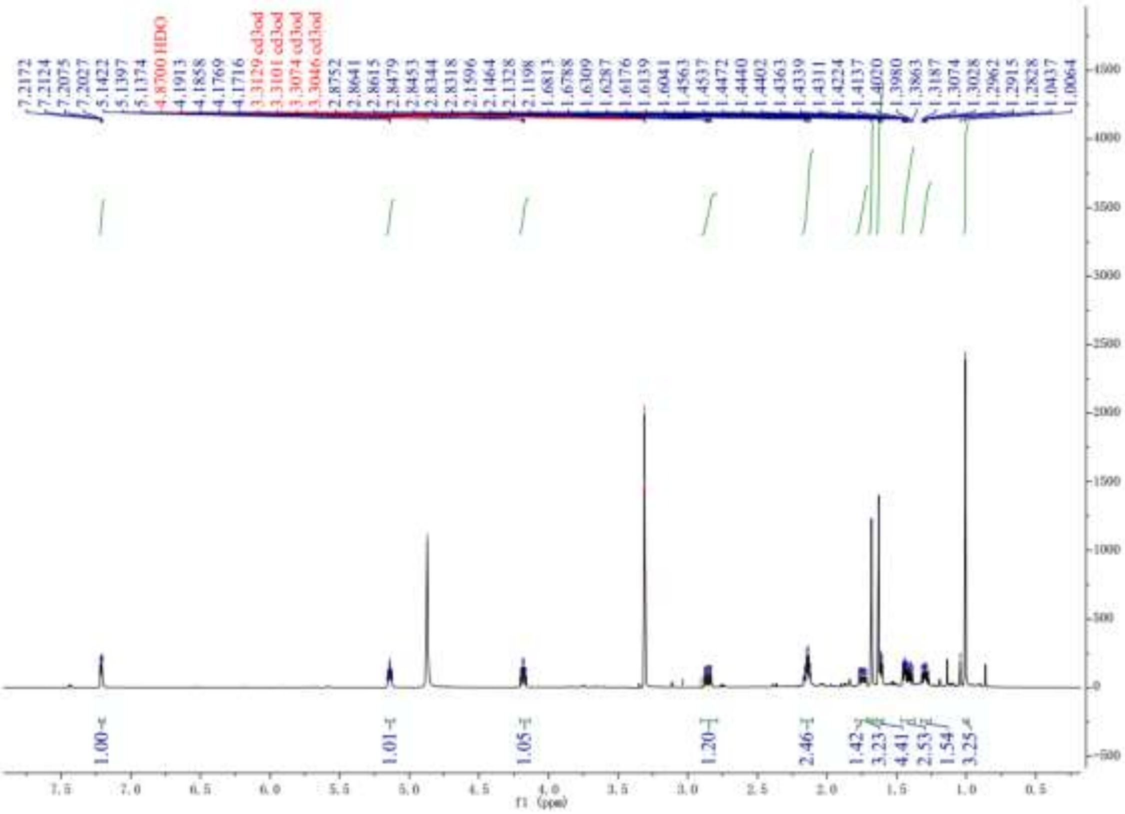

Figure S27. ^1H NMR (600 MHz, CD_3OD) spectrum of compound **3**.

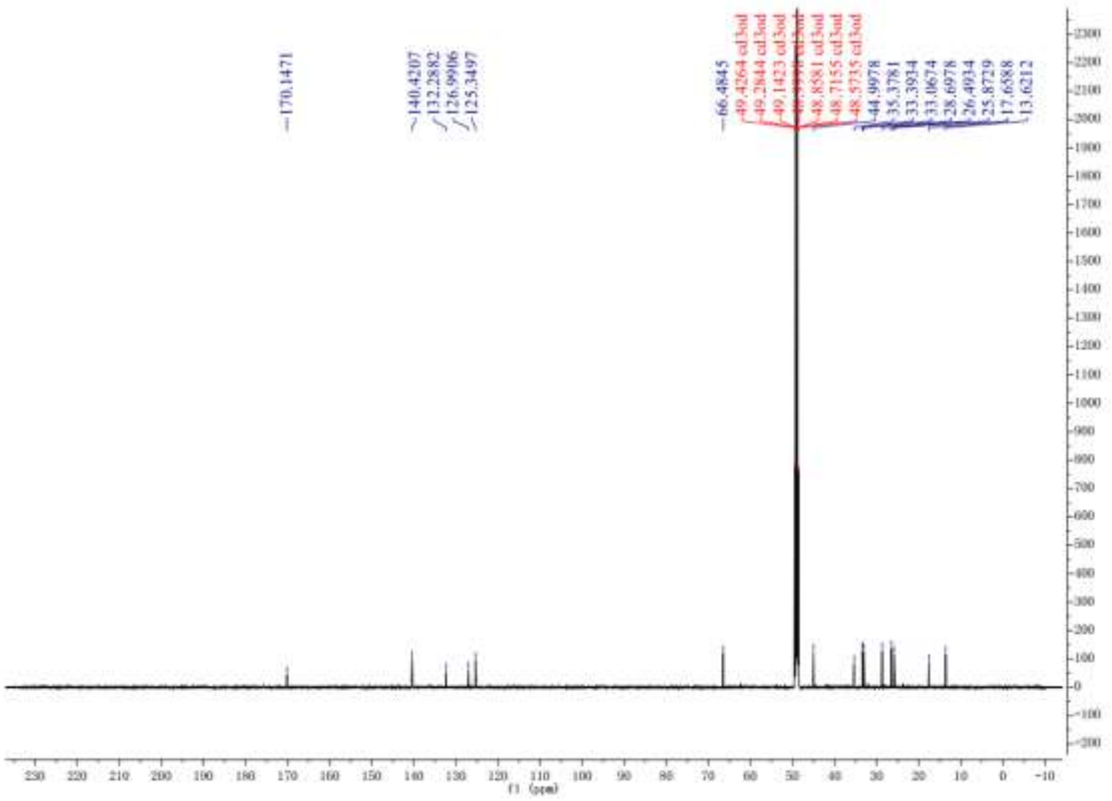

Figure S28. ^{13}C NMR (150 MHz, CD_3OD) spectrum of compound **3**.

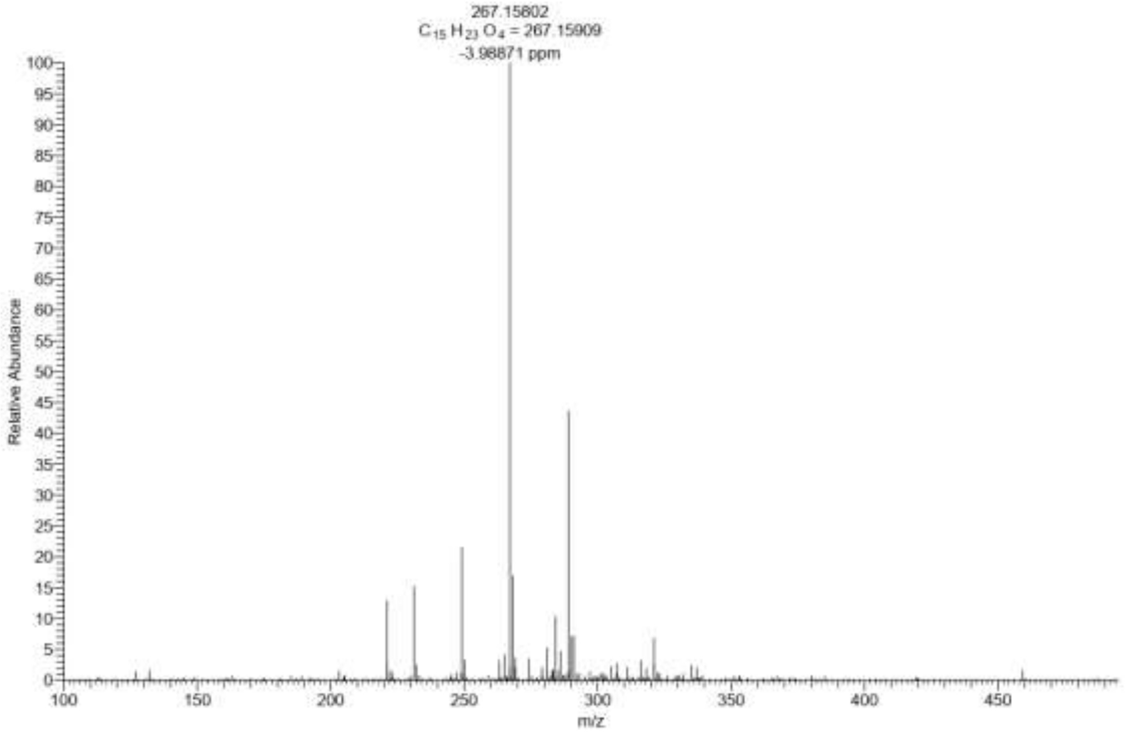

Figure S29. HR ESIMS spectrum of compound **5**.

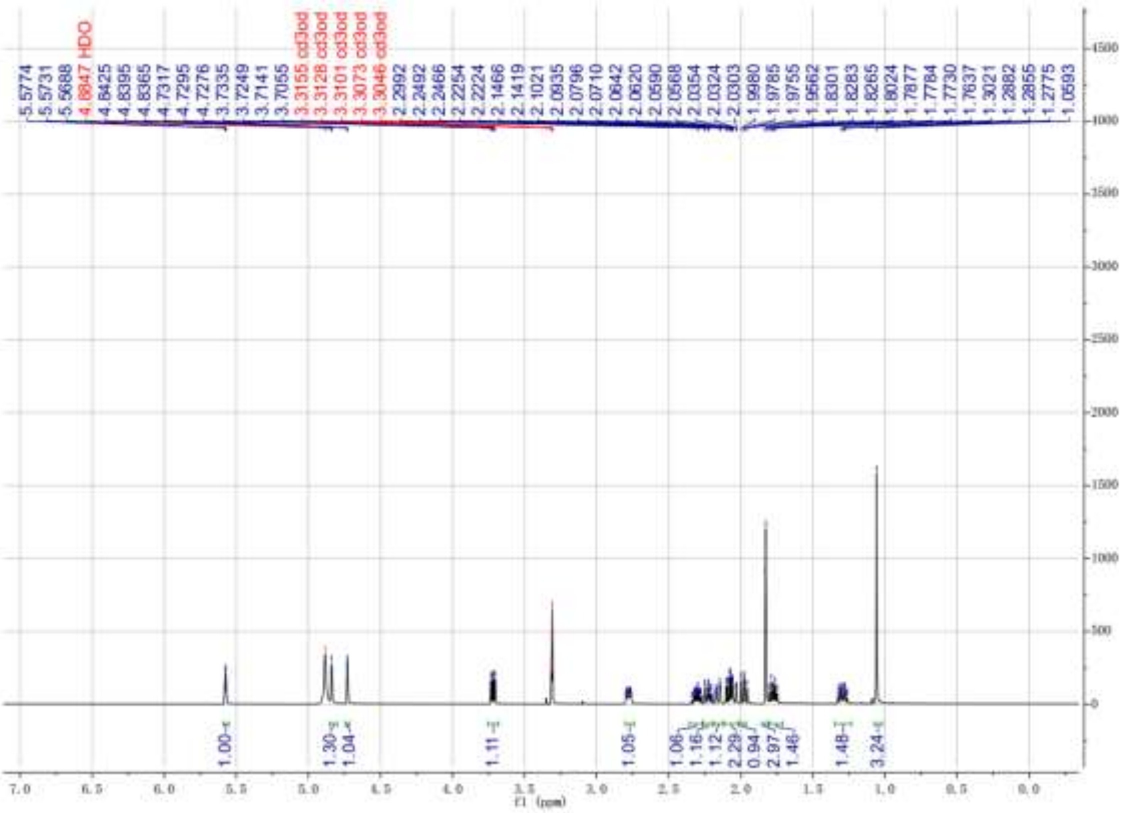

Figure S30. ^{1}H NMR (600 MHz, CD$_3$OD) spectrum of compound **5**.

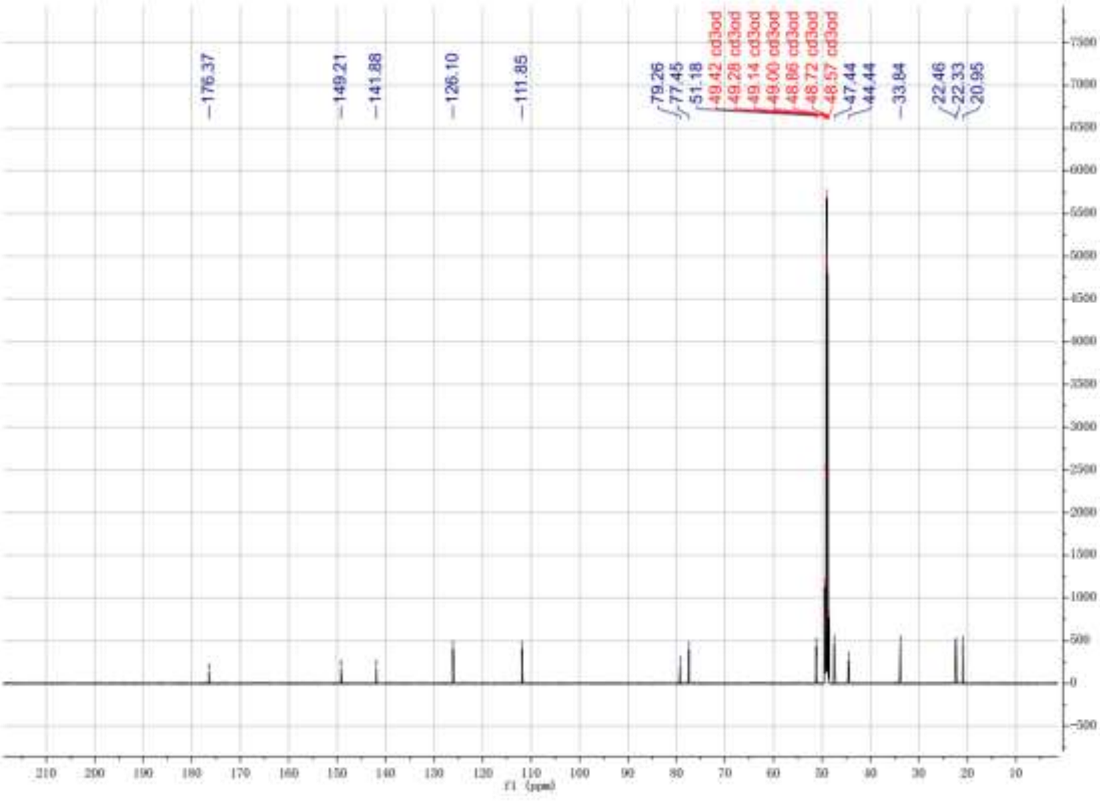

Figure S31. ^{13}C NMR (150 MHz, CD$_3$OD) spectrum of compound **5**.

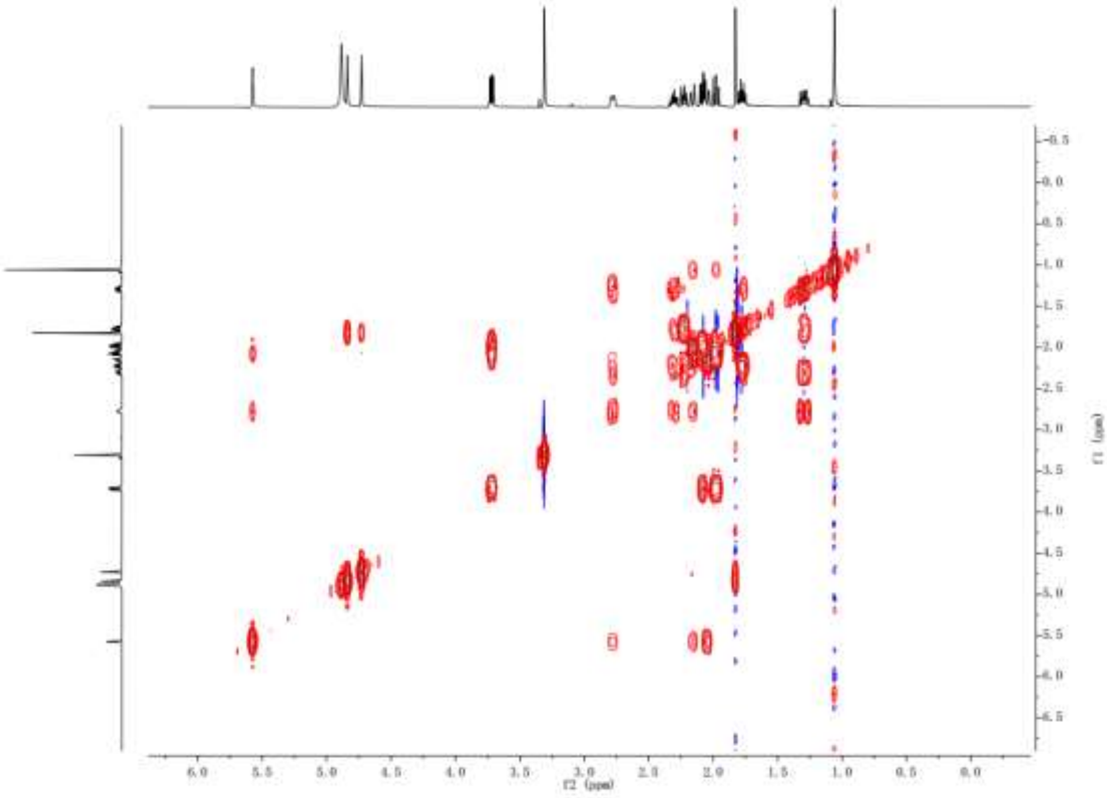

Figure S32. ^{1}H-^{1}H COSY (600 MHz, CD_3OD) spectrum of compound **5**.

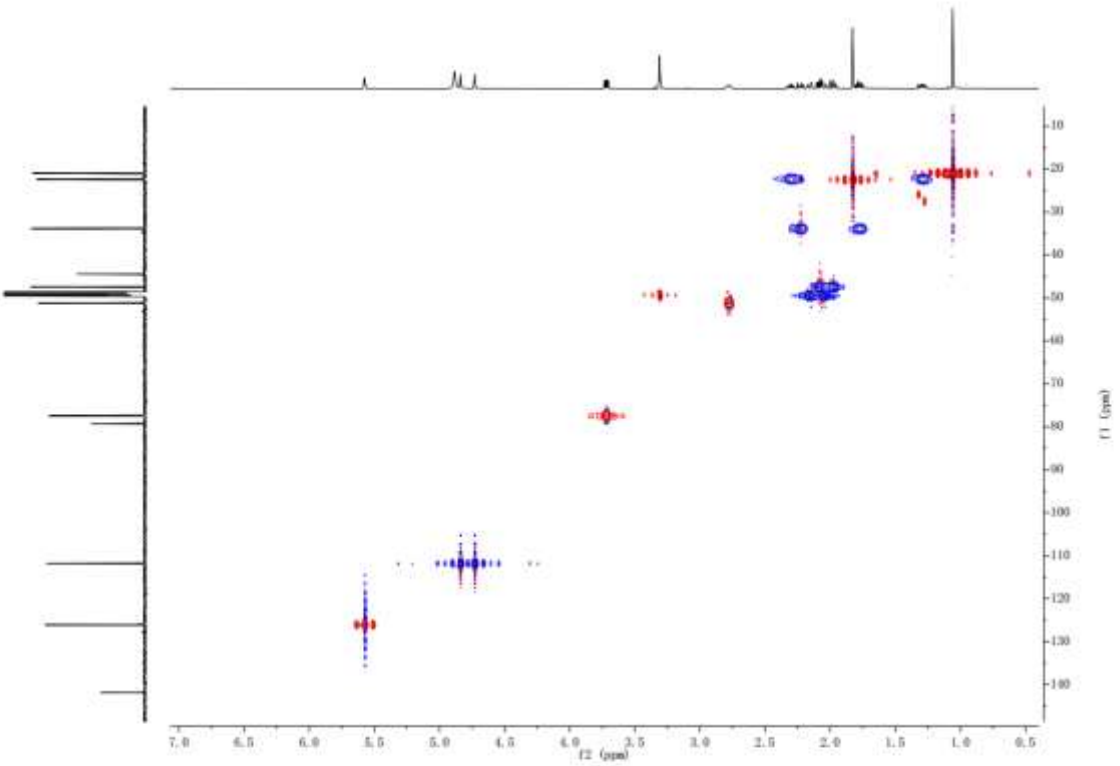

Figure S33. HSQC (150 MHz, CD_3OD) spectrum of compound **5.**

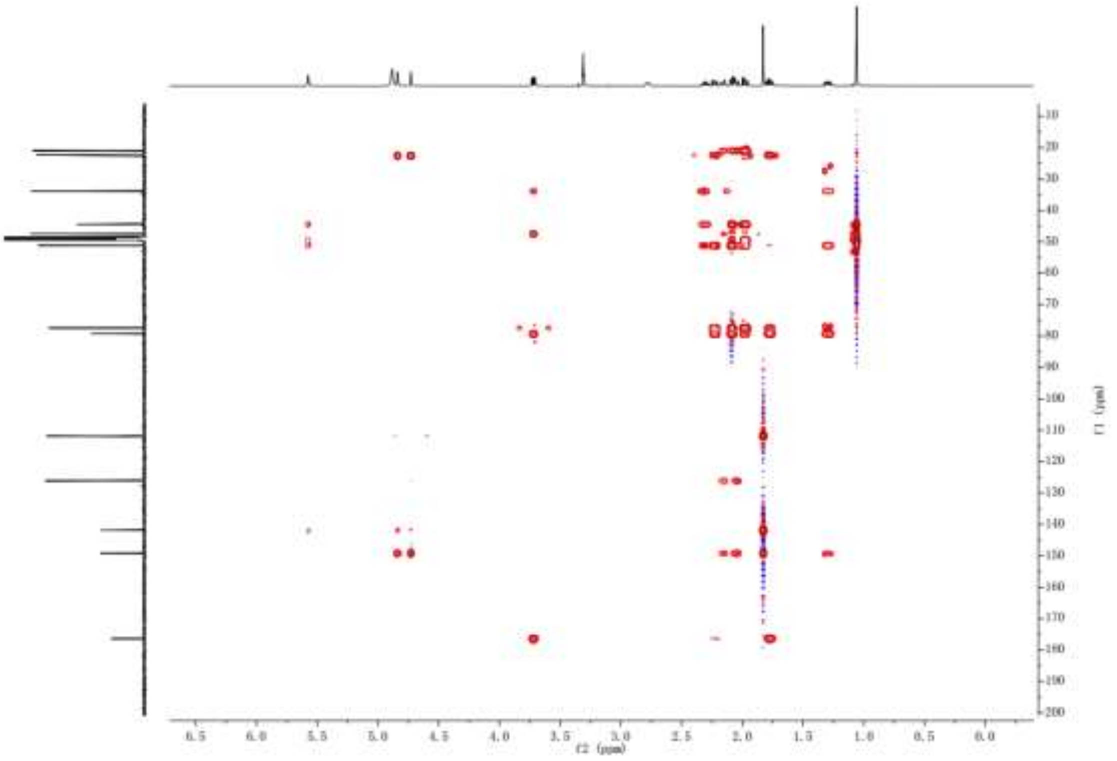

Figure S34. HMBC (150 MHz, CD_3OD) spectrum of compound **5**.

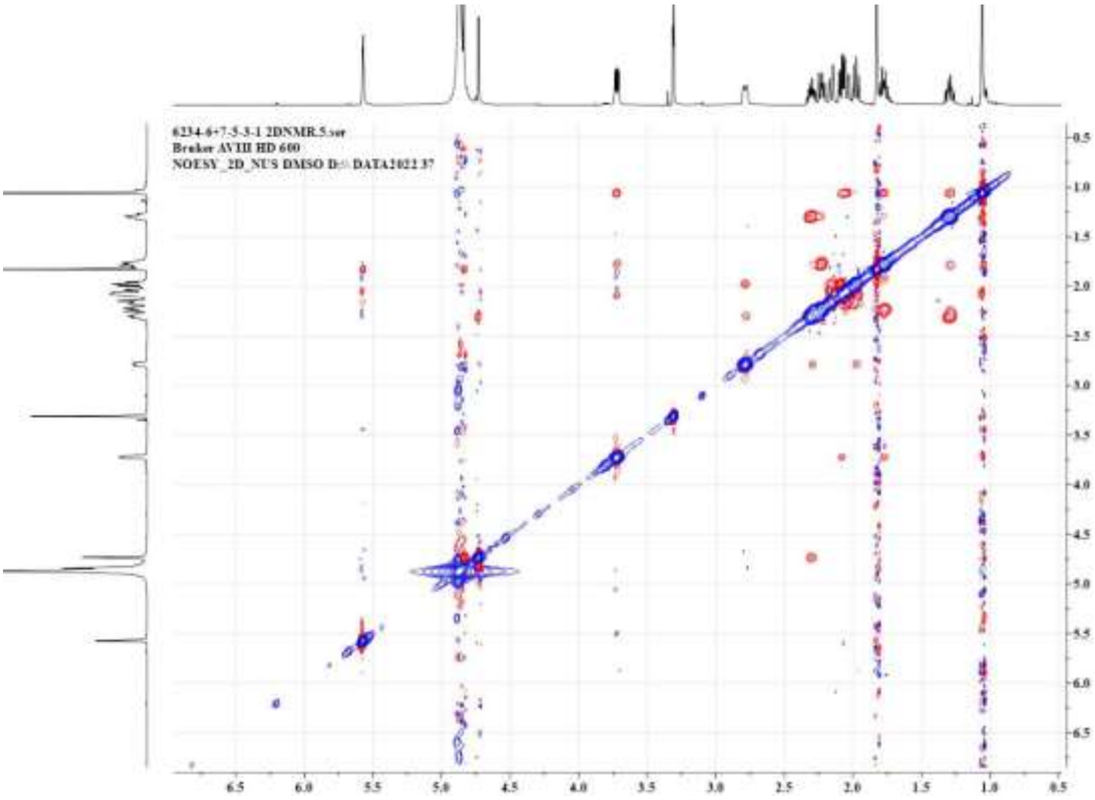

Figure S35. NOESY (600 MHz, CD_3OD) spectrum of compound **5.**

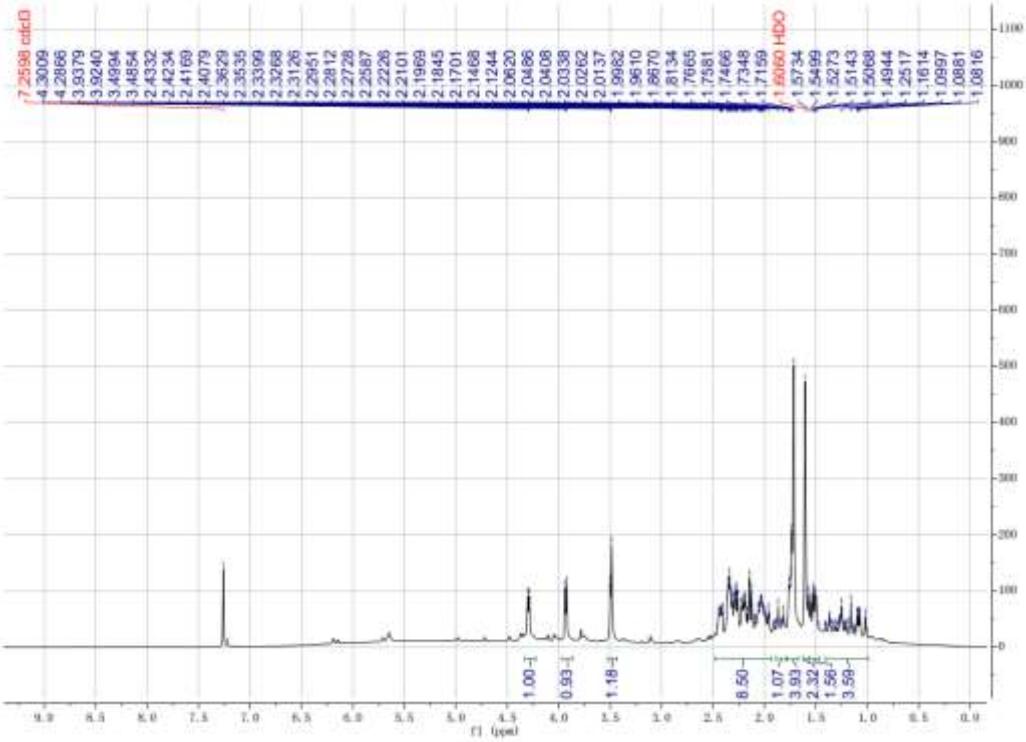

Figure S36. ^{1}H NMR (600 MHz, $CDCl_3$) spectrum of compound **6**.

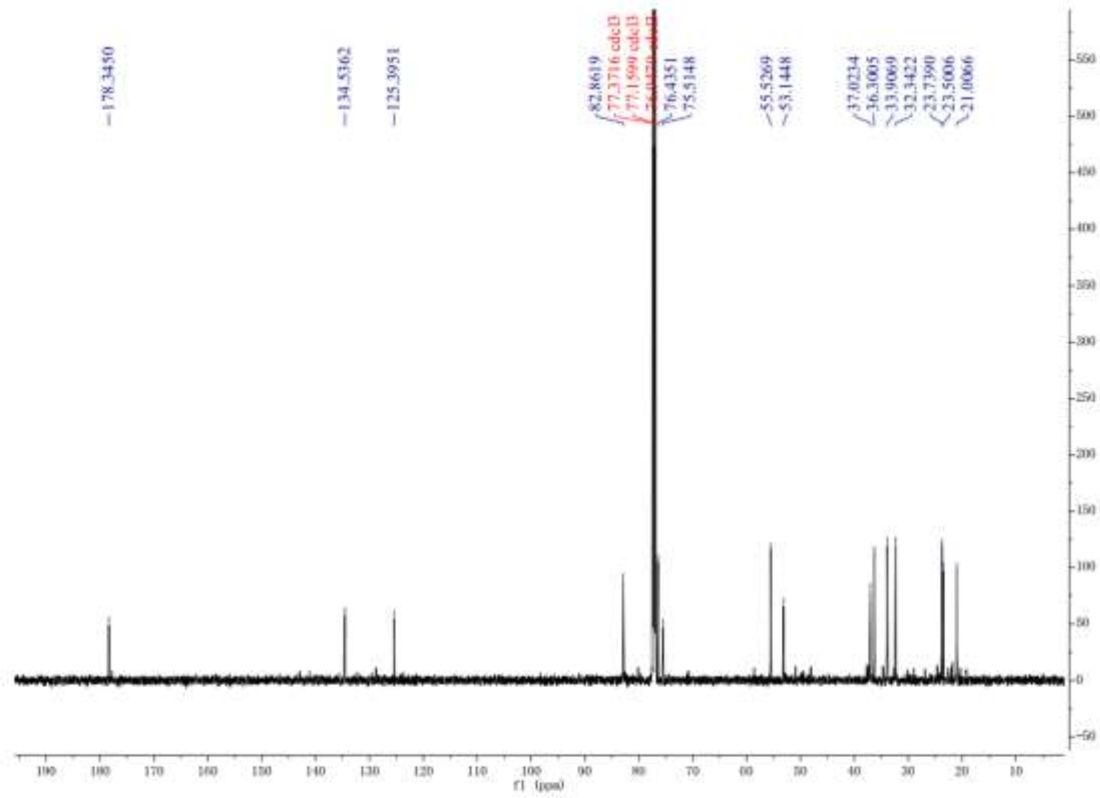

Figure S37. ^{13}C NMR (150 MHz, $CDCl_3$) spectrum of compound **6**.
